# Disequilibrium of fire-prone forests sets the stage for a rapid decline in conifer dominance during the 21^st^ century

**DOI:** 10.1038/s41598-018-24642-2

**Published:** 2018-04-30

**Authors:** Josep M. Serra-Diaz, Charles Maxwell, Melissa S. Lucash, Robert M. Scheller, Danelle M. Laflower, Adam D. Miller, Alan J. Tepley, Howard E. Epstein, Kristina J. Anderson-Teixeira, Jonathan R. Thompson

**Affiliations:** 1 Harvard Forest, Harvard University, Petersham, MA USA; 2 Section of Ecoinformatics and Biodiversity, Department of Bioscience, Aarhus University, Ny Munkgade 116, 8000 Aarhus C, Denmark; 3 Center for Biodiversity Dynamics in a Changing World (BIOCHANGE), Department of Bioscience, Aarhus University, Ny Munkegade 114, DK-8000 Aarhus, Denmark; 4 UMR Silva, AgroParisTech, Université de Lorraine, INRA, 54000 Nancy, France; 5 Dept. Forestry and Environmental Resources, North Carolina State University, Campus Box 7106, Raleigh, NC 27695 USA; 6 Department of Geography, Portland State University, P.O. Box 751, Portland, OR 97207 USA; 7 Conservation Ecology Center, Smithsonian Conservation Biology Institute, Front Royal, Virginia USA; 8 Department of Environmental Sciences, University of Virginia, Charlottesville, VA USA; 9 Smithsonian Tropical Research Institute, Panama, Panama

## Abstract

The impacts of climatic changes on forests may appear gradually on time scales of years to centuries due to the long generation times of trees. Consequently, current forest extent may not reflect current climatic patterns. In contrast with these lagged responses, abrupt transitions in forests under climate change may occur in environments where alternative vegetation states are influenced by disturbances, such as fire. The Klamath forest landscape (northern California and southwest Oregon, USA) is currently dominated by high biomass, biodiverse temperate coniferous forests, but climate change could disrupt the mechanisms promoting forest stability (e.g. growth, regeneration and fire tolerance). Using a landscape simulation model, we estimate that about one-third of the Klamath forest landscape (500,000 ha) could transition from conifer-dominated forest to shrub/hardwood chaparral, triggered by increased fire activity coupled with lower post-fire conifer establishment. Such shifts were widespread under the warmer climate change scenarios (RCP 8.5) but were surprisingly prevalent under the climate of 1949–2010, reflecting the joint influences of recent warming trends and the legacy of fire suppression that may have enhanced conifer dominance. Our results demonstrate that major forest ecosystem shifts should be expected when climate change disrupts key stabilizing feedbacks that maintain the dominance of long-lived, slowly regenerating trees.

## Introduction

Climate change is increasingly altering the species composition and carbon (C) sequestration of forest landscapes. These changes may have a profound effect on local and regional biodiversity and they could produce a positive carbon-cycle feedback to climate change when high-biomass forests are converted to lower-biomass, shrub-dominated ecosystems. Yet the magnitude and rate of these changes and how they will interact with land-use legacies is not well understood. Statistical associations between species distributions and climate suggest the potential for rapid changes in forest species ranges^1,2^. Conversely, mounting evidence suggests forest communities often experience lagged responses to climate change^3–5^, which are commonly characterized as “debts” (or “borrowed time”^6^). Climatic debt accrues when species do not fully track changes in their climatically suitable environment^3^; resilience debt accrues when species are not adapted to a given disturbance regime and are therefore susceptible to extirpation following those disturbances^7^. The presence of ecological debts suggests that forest communities are in a climatic disequilibrium, but predicting the timing or magnitude of community shifts in the presence of such debts, together with climate change, is challenging and an urgent priority for global change ecology.

**Figure 1:**
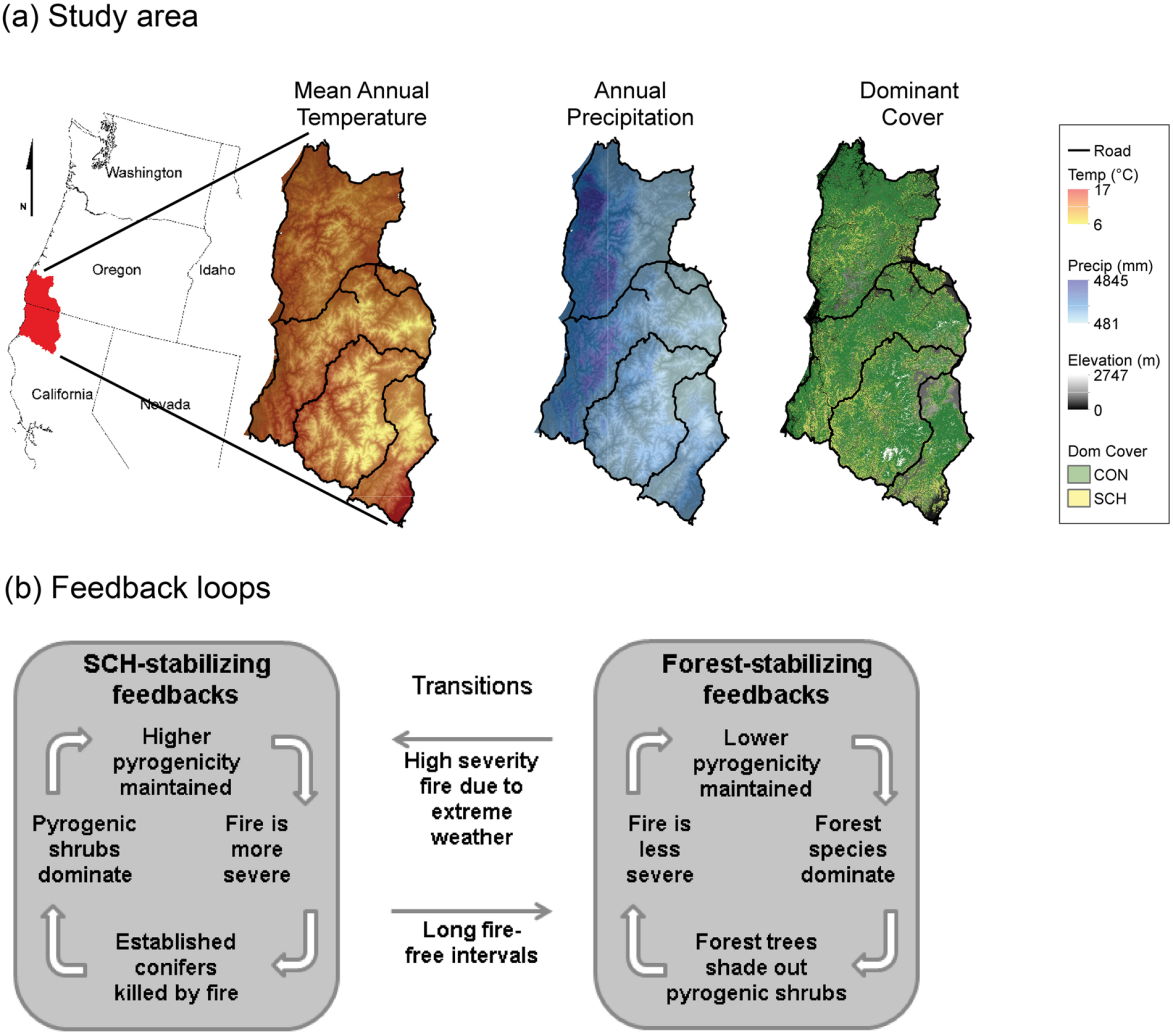
The Klamath forest landscape biome transition and forest dynamic feedbacks triggering transitions. (**a**) Study area temperature, precipitation, and initial forest dominant type (left to right), and (**b**) feedbacks that maintain the two forest community states (adapted from^24^). CON represents conifer forest community states, SCH represent shrubland-chaparral-hardwood community state. Maps were created using ArcGIS v.10.5 (www.ersi.com/argis).

In fire-prone temperate forests, stable community states are often maintained via feedbacks, such as when species’ characteristics reinforce a specific fire regime (e.g. Fig. 1a,b plus see other feedbacks between vegetation and fire^8,9^). When exogenous drivers such as climate change alter a fire regime, the feedbacks that maintain stable communities can break down and vegetation can shift rapidly. Indeed, when climate change alters disturbance regimes it can result in much faster changes to vegetation communities than those that result from shifts in competitive abilities between species (or other biotic interactions) due to climate change, which occur over much longer temporal scales in forests (>100 years).

Climate change is expected to increase the occurrence, extent, and severity of fires in forested systems^10,11^. These alterations could be driven largely by higher temperatures with subsequent potential effects such as increased ignitions, longer fire seasons and faster fire spread rates due to decreased fuel moisture^12,13^. Furthermore, higher temperatures may delay forest development after a fire, given that both recruitment and growth may be hampered in more arid environments^14–19^. These responses threaten the persistence of self-sustaining feedbacks that maintain forest cover (Fig. 1) and thus the resilience of forests to fire, which has become a major concern in fire-prone temperate forests^7,17,20,21^.

Here we investigate the potential for rapid (<100 years) and large-scale transitions in forest communities in the Klamath forest landscape (northern California and southern Oregon, USA). We explore the potential for shifts between high biomass coniferous temperate forests and lower biomass Mediterranean sclerophyllous shrub, chaparral, and hardwood communities (hereafter *shrubland-hardwoods*; Fig. 1). The Klamath forest landscape (hereafter, Klamath) is a major carbon reservoir and an internationally recognized hotspot of botanical biodiversity^22,23^ (Fig. 1a). The conifers and shrubland-hardwood communities are thought to function at local scales as alternative stable states, due to their self-stabilizing feedbacks involving biotic and climate-fire interactions^24–26^ (Fig. 1b). There have been long-standing concerns regarding conifer regeneration failure in the Klamath, originally associated with post clear-cut logging environments^27^, but in recent decades associated with the aftermath of high severity wildfire^17^. The shrubland-hardwood state is composed of highly pyrogenic species^28^, many of which sprout vigorously after fire and inhibit conifer regeneration^27^. Consequently, the shrubland-hardwood communities promote a self-reinforcing fire regime^29^. Only when the fire-free interval is sufficiently long (as often occurs when fire is suppressed) can conifers overtop the shrub layer and begin to dominate the community^30^. For most conifer species in the Klamath (e.g., *Pseudotsuga menziesii* and *Calocedrus decurrens*), the ability to survive fires is based on adaptations to fire that are more apparent in older trees (e.g. bark thickening, increasing crown base height^8,30^). They eventually grow large enough to survive surface fires that primarily affect the understory and inhibit ladder fuel development, and thereby reinforce conifer dominance^9^.

Observations from the Klamath offer an important case study with implications for other fire-prone forests. Because large-scale experimentation in forests is unrealistic, mechanistic models with explicit representations of species and their adaptations to wildfire offer a useful tool to explore individual and interacting effects of climate change drivers as well as feedback mechanisms. Additionally, mechanistic models allow consideration of a breadth of spatial and temporal scales in forest community dynamics. Here, we evaluate the hypothesis that a warming climate and increased fire activity will drive rapid conifer decline in the Klamath, in favor of shrubland-hardwood. We expected that, compared to forest dynamics driven by the current climate (baseline conditions; 1949–2010), climate change would (1) reduce fire rotation period, (2) slow conifer growth and hamper conifer regeneration compared to shrubland-hardwood, and (3) trigger major reductions in the area of conifer dominance, allowing for the replacement of shrubland-hardwood community types.

## Results

### Climate change impacts on forest regeneration and growth

**Figure 2:**
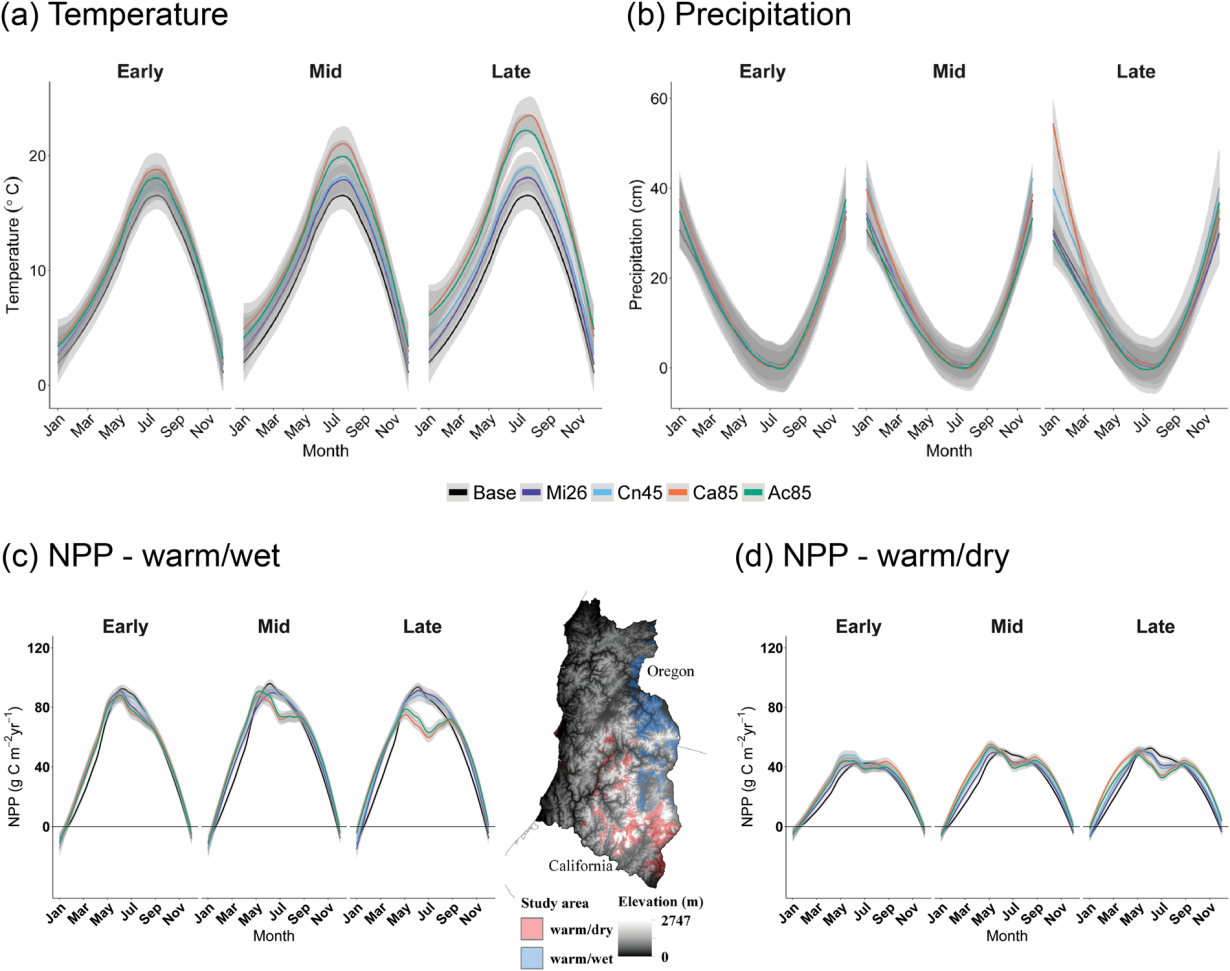
Climate seasonal regimes and the simulated effects on forest productivity. (**a**) Temperature and (**b**) Precipitation under baseline and climate change conditions for the Klamath landscape. Effects of climate on forest net primary productivity (NPP) in two locations chosen to highlight the model’s response to environmental gradient under all climate scenarios: (**c**) warm-wet and (**d**) warm-dry. See Table 1 for climate change scenario acronyms. Time-periods: Early 2015–2042, Mid 2043–2070, Late 2071–2100. Maps were created using ArcGIS v.10.5 (www.ersi.com/argis).

Over the next century, the projected increase in annual mean temperature ranged from 1.2 to 2.9 °C across the four future scenarios (Fig. 2a). The rate of temperature and precipitation change increased over the simulation period, thus the differences among scenarios were more evident towards the latter half of the 21^st^ century. Climatic differences varied seasonally, with the greatest increases in spring and fall (RCP 8.5; Fig. 2). Also, summers were projected to be hotter –ranging from +1.2 to +4.9 °C/month. Projected precipitation also varied seasonally and among scenarios (Fig. 2b). Winter precipitation ranged from a 2.24 mm/month decrease to a 13.16 mm/month increase across scenarios by the end of the century. In contrast, all scenarios projected a summer precipitation decrease between 12% and 61% at the end of the century (see Table 1 for scenario categorization).

**Table 1:** Baseline and climate change scenario projections. Relative projections are a qualitative description of Fig. S1 offered here to assist with synthesis. These categories are based on average annual statistics over the course of the simulation (85 years) for mean annual temperature and annual precipitation. Annual probability of establishment shifts across species in conifer and shrubland-chaparral-hardwood (SCH) species under different climatic conditions. These values are averages across species and time.

Climate Scenario	Emissions scenario (RCP)	Climate model	Relative projections^*^	Fire Rotation Period^*1^	Median Fire sizeb (ha)	Total burned area in large high severity patches >50 ha (×1,000 ha)	Persistent shift from conifer to hardwood-chaparral (×1,000 ha)	Annual establishment probability*^2^	
								Conifers	SCH
Baseline (1949–2010)	na	na	na	108 [104–123]	5,091	448	580	0.23	0.30
Mi26	2.6	MIROC5	Mild hot – wetter	114 [98–117]	5,029	498	580	0.17 (26%)	0.28 (7%)
Cn45	4.5	CNRM-CM5	Hotter – wetter	108 [91–116]	5,567	529	613	0.18 (22%)	0.29 (3%)
Ac85	8.5	ACCESS	Much hotter - drier	89 [82–100]	6,314	582	622	0.15 (35%)	0.25 (17%)
Ca85	8.5	CanESM2	Much hotter - wetter	91 [82–99]	6,102	594	606	0.14 (39%)	0.24 (20%)

^*^See Fig. S1 for quantitative values.

*^1^Range in brackets indicates 25^th^–75^th^ percentile.

*^2^Percentages in parenthesis indicate the percentage of probability of establishment loss with respect to baseline conditions.

Modeled forest growth rates varied spatially along gradients of soil-water availability, as driven by variation in precipitation, temperature, and physical soil characteristics (Fig. 2c and d). To better understand changes in growth rates, we highlight two ecoregions that bound the gradient of water balance conditions in the area, and thus show different responses to environmental conditions. In these ecoregions, modeled monthly cohort net primary productivity for all species present ranged between 0.11 g C m^−2^ mo^−1^ and 108.9 g C m^−2^ mo^−1^ under different scenarios. The model projected shifts in growth, with strong growth limitation during summer under climate change (Fig. 2c and d), with decreases of up to 22 gC m^−2^ yr^−1^ in July in wetter climates (late Ca85 scenario; Fig. 2c) and 11 gC m^−2^ yr^−1^ in July in drier climates (late Ac85 scenario; Fig. 2d). The model also projected a lengthening of the growing season, with the greater growth enhancement in late winter/early spring (Fig. 2c and d). For instance, growth could potentially increase by 25 gC m^−2^ yr^−1^ and 11 gC m^−2^ yr^−1^ in March in the high forcing scenarios (late Ca85 and Ac85 scenarios). Across all climate change scenarios, the trend of earlier onset of growth in winter-spring and reduced growth in summer became more pronounced later in the 21^st^ century (Fig. 2).

Post-fire establishment probability for all species was reduced under climate change due to increased summer drought (Table 1). Depending on the climate change scenario, the decline ranged from 26 to 39% for conifer species and 7 to 20% for the shrubland-hardwood group (Table 1). In general, the probability of establishment was lower for the higher temperature forcing scenarios (Ac85 and Ca85 versus Mi26 and Cn45 scenarios). The probability of establishment was higher in Mi26 vs Cn45, compared to other climate change scenarios, because water availability was higher in Cn45 than in Mi26 (Table 1 and Fig. S1).

### Climate change impacts on fire activity

**Figure 3:**
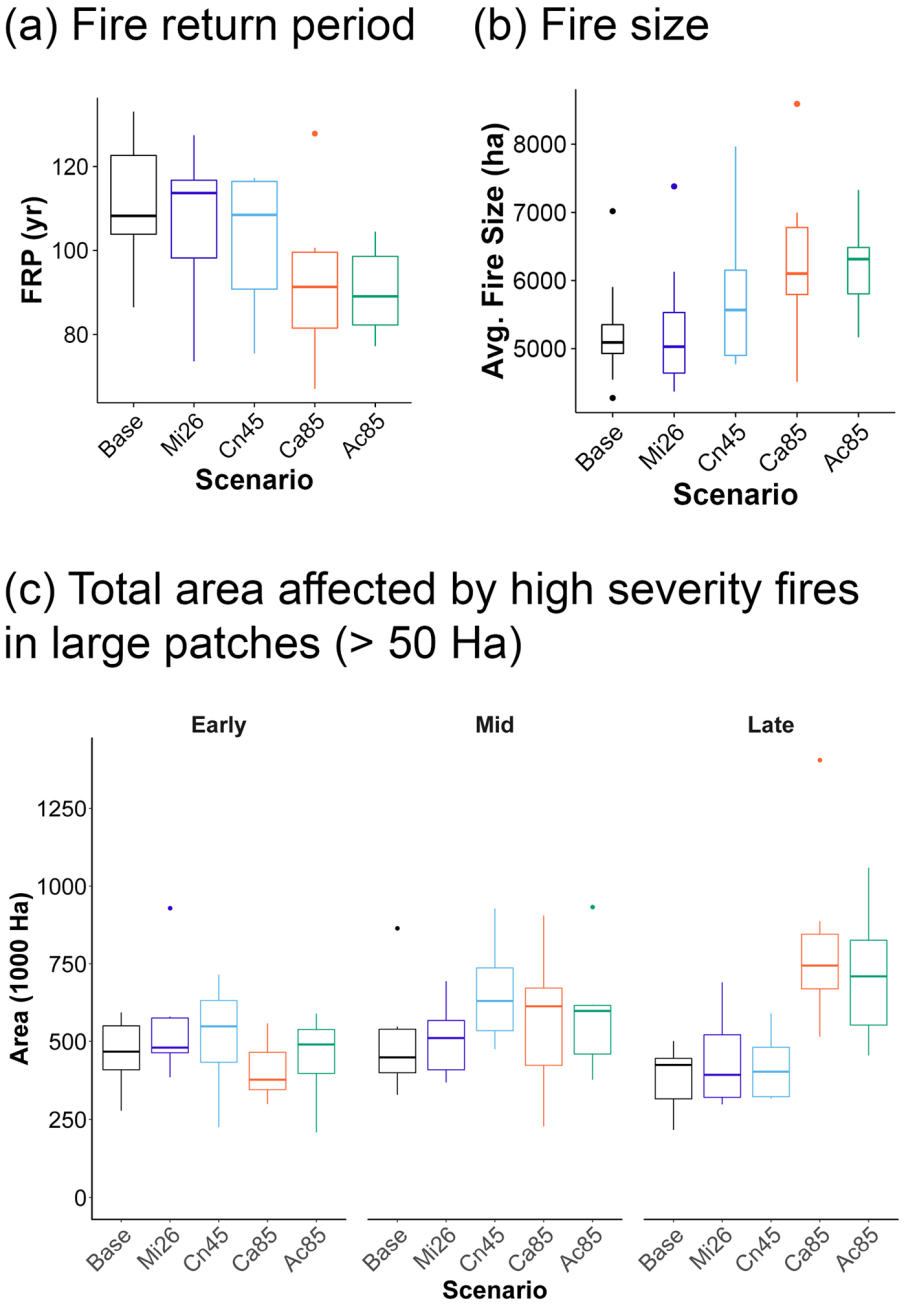
Fire regime model outputs. (**a**) Fire return period – time to burn an area of the same size of the area of study; (**b**) Average fire size for different simulation repetitions under baseline and climate change scenarios; (**c**) Total area of high severity fire for different scenarios; (**d**) High severity area change for large fire patches (>50 ha). Boxplot represents different the distribution of values across 9 simulation repetitions. See Table 1 for climate change scenario acronyms.

Climate change was associated with greater fire activity in all scenarios (Fig. 3; Table 1). Fire rotation periods (FRP; number of years needed to burn an area the same size as the study area) were reduced by 19 and 17 years for the highest forcing climate change scenarios (i.e. Ca85 and Ac85 scenarios, Fig. 3a and Table 1), whereas there was an increase (5 years) in the FRP under Mi2.6 and no difference in the Cn4.5 scenarios. There was high variability among simulations within each climate change scenario, especially for those scenarios with the least forcing. Average FRP (across simulation replications) differed significantly from the baseline conditions after 85 years in the case of the hottest-dry scenario (Ac85; Fig. 3a, Supplementary Table S1).

Average fire size was larger under the climate change scenarios (Fig. 3b), particularly the warmest scenarios (Ca85 and Ac85 scenarios). The model projected a maximum average increase of 1,011 ha and 1,222 ha between baseline conditions and Ca85 and Ac85 scenarios, respectively (Table 1). This resulted in average fire sizes of 6,102 (Ca85) and 6,314 ha (Ac85) (Table 1 and Fig. 3b) that did not significantly differ from baseline conditions (Supplementary Table S2). More importantly, the area of high severity fire in large patches (>50 ha) increased under the climate change scenarios (Fig. 3c), hampering a rapid recolonization of new tree recruits (further distance from seed sources). The area of these patches increased rapidly in the latter part of the century for the high forcing scenarios, with median values of area burnt at high severity between 744,000 ha (Cn85) and 710,000 ha (Ac85). This represents a significant increase of 320,000 ha and 285,000 ha with respect to the baseline historic conditions (Table 1 and Supplementary Table S3).

**Figure 4:**
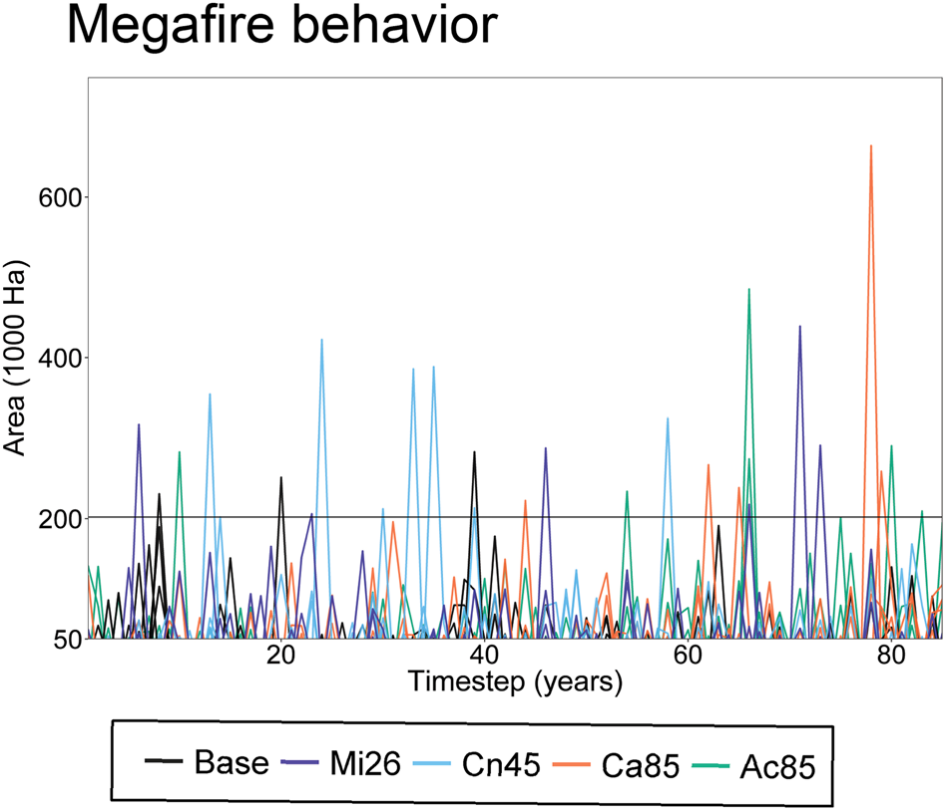
Time series of maximum fire size for different model simulation. Horizontal solid line indicates the historical maximum fire size recorded in the area (Biscuit fire 202,000 ha). See Table 1 for climate change scenario acronyms.

Very large fires in the warmest climate change scenarios (Ac85 and Ca85 scenarios) ranged between 200,000 ha and 500,000 ha in several years (Fig. 4), breaking the record of the largest recorded fire in the region and the baseline simulations (200,000 ha, solid horizontal line in Fig. 4). Only in one of the highest forcing scenarios simulations did a fire exceed 500,000 hectares (1 out of 9 repetitions per scenario under Ca85 and Ac85, Fig. 4).

**Figure 5:**
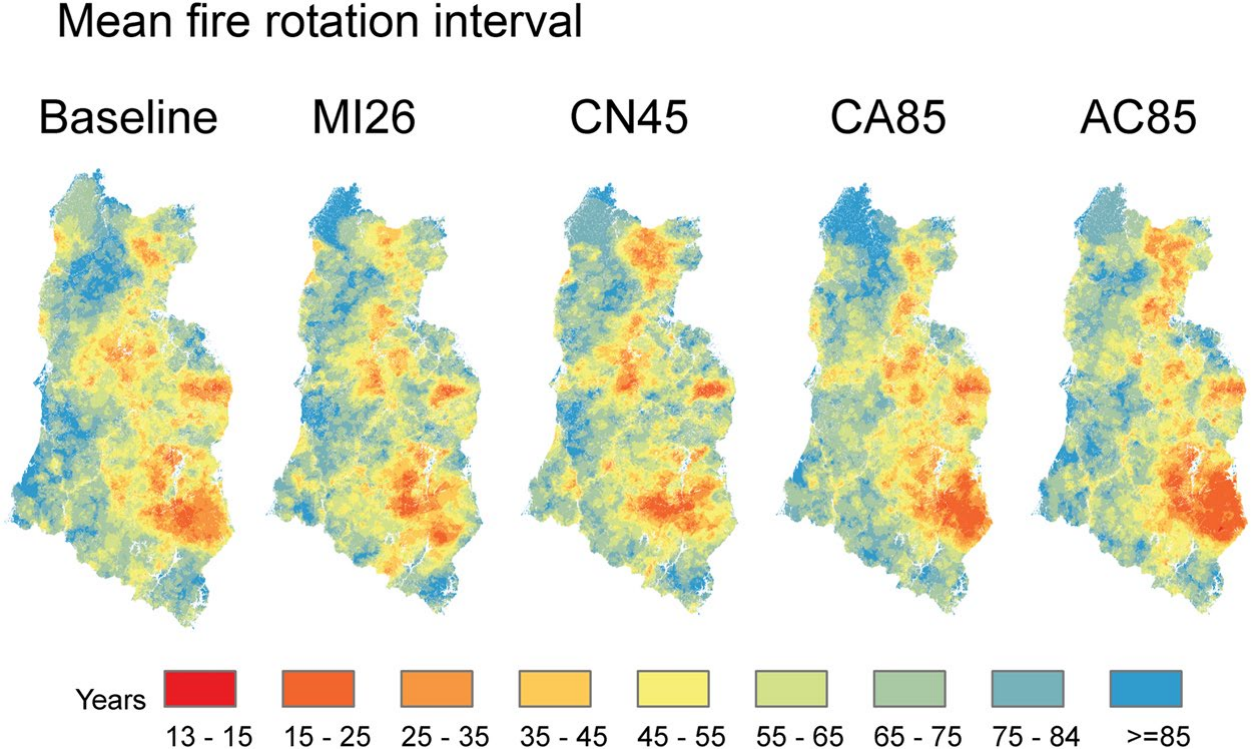
Spatial distribution of mean fire return intervals (MFRI) in the area. MFRI above 85 indicates that no fire was recorded in the area for the simulations analyzed. See Table 1 for climate change scenario acronyms. Maps were created using raster package v 2.3.40 in R 3.3.0 (https://www.r-project.org/).

Shorter fire return intervals were more prevalent in the eastern side of the study area and ranged between 13 and 25 years (Fig. 5). This pattern was consistent with the west-to-east gradient of increasing water deficit (Fig. 1a). The total area with mean fire return intervals under 25 years only increased by 1–5%, depending on the climate change scenario (Fig. 5).

### Dominance shifts result from changes in growth rates, establishment, and fire regimes. 

**Figure 6:**
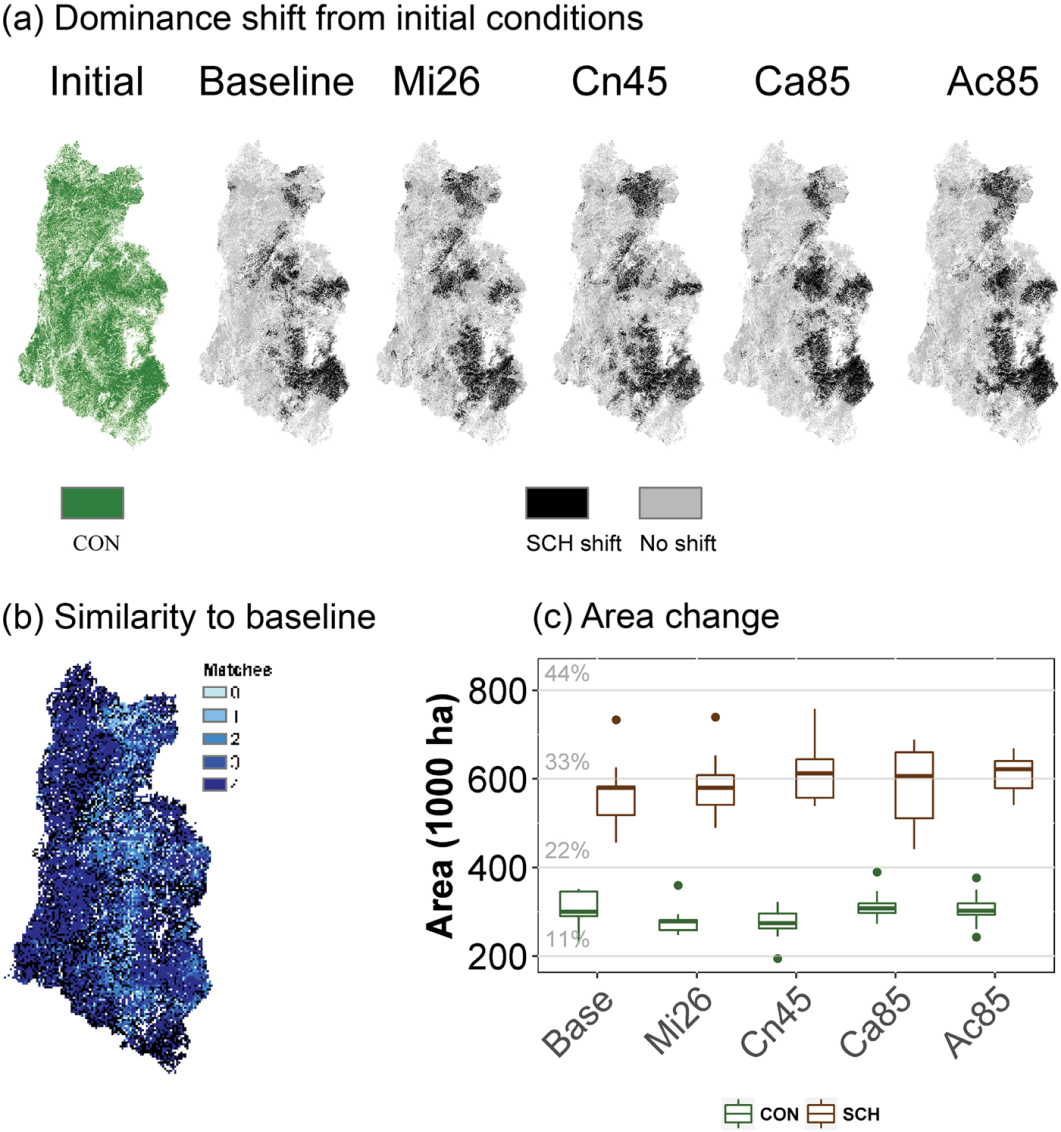
Shifts in forest type. (**a**) Forest dominance shifts compared to initial conditions. (**b**) Similarity index between four climate change scenarios and baseline conditions. The index describes how many of the climate change scenarios agree with the baseline scenario (4 = maximum agreement, 0 = maximum disagreement); and (**c**) Area of conifer forest transitions remaining as conifer (CON) or shifted to shrubland-hardwood (SCH). Dominance shift from CON to SCH was calculated when SCH dominance was persistent for more than 30 years See Table 1 for climate change scenario acronyms. Maps were created using raster package v 2.3.40 in R 3.3.0 (https://www.r-project.org/).

Relative to current vegetation patterns, simulations of both baseline and climate change conditions resulted in large, persistent shifts in vegetation dominance from conifers to shrubland-hardwood, particularly in the drier eastern half of the study area (Fig. 6a, Table 1). The model estimated the loss of conifer dominance on more than 500,000 ha during 85 years of simulation, even under current climate conditions (e.g. baseline; dominance loss 580,000 ha, Table 1), approximately 31% of the current conifer forest extent in the study region. We defined vegetation dominance shifts as conversions from conifer forest to shrubland-hardwood that persist >30 years to the end of the simulation (2100) and thus exclude conversions that recovered to forest by the end of the simulation, and conversions to shrubland-hardwood that occurred toward the end of the simulation and might not be persistent. Major shifts were located where the model projected high fire activity in the first half of the century and where conifers were never able to reassert dominance. The most pronounced spatial disagreement between baseline and climate change simulations was concentrated in the center of the study area, where higher elevations and greater topographic complexity is present (Fig. 6b).

Climate change scenarios range from almost indistinguishable changes in the area of conifer forest lost between climate change and baseline – both baseline and Mi26 conifer loss of 580,000 ha) and 622,000 ha in the case of the hot-dry scenario (Ac85; Fig. 6c and Table 1). Climate change scenarios that presented higher precipitation resulted in greater intra-scenario variation in fire regimes (Fig. 6c, Cn45 and Ca85).

## Discussion

Our results demonstrate the potential for rapid decline of conifer dominance in the Klamath during the 21^st^ century. These results supported our initial predictions, except that these changes also occurred in the absence of climate change. The area undergoing shifts to shrubland-hardwood increased when simulations included climate change scenarios, due to a reduction in the fire rotation period (Fig. 3a), an increase in fire size and severity (Fig. 3b,c), a reduction in forest growth (Fig. 2), and a decline – especially for conifers—in establishment (Table 1). The results of our simulations support the hypothesis that climate change will result in major reductions in conifer forest dominance, concurring with studies from other mixed conifer and subalpine forests in the western United States that showed or predicted major vegetation shifts^15,31^.

### Shifts in growth, establishment, and fire under climate change lead to conifer dominance decline

The simulations projected increased fire size under climate change scenarios, concurring with other empirical work in western US forests^13,32^. Particularly interesting is that the model suggested a potential increase in large fires (>50,000 ha) under climate change, surpassing the historical maximum fire sizes recorded in the area (e.g. Biscuit Fire 202,000 ha; Fig. 4), in accordance with other projections based on statistical correlations in a similar ecological zone in northern California^11^. Interestingly, both fire size and severity differ from baseline values by the end of the 21^st^ century. The ‘slightly wetter’ scenario (Ca 85) produced similar, albeit highly variable, fire activity in terms of size and severity (Fig. 3) to the ‘slightly drier’ scenario (Ac85). This implies that scenarios of climate change may be wetter on a yearly temporal scale due to spring or winter rain, but drier summers may still promote higher fire activity.

The model projected an overall reduction in annual tree cohort growth, although there was high variation, dependent on the species, climate, and soils (see growth related parameters in Tables S4 and S5). Overall, and particularly for the drier portions of the study area, summer growth is projected to decline; this finding is consistent with the results from a study of tree-ring chronologies in the region^33^. Conversely, the model suggests a potential for increased growth in the spring and to a lesser extent in the fall (Fig. 2b; see also^34^). Indeed, extended growing seasons could increase growth and thus the ability of conifers to recover after a fire (see^35^ and references therein), especially for the higher elevations in our study region. Using combined flux tower measurements and satellite information, it has been found that an earlier start to the growing season could have reduced the impact of a subsequent year summer drought in 2012^36^. In contrast, our model output suggests that the increased spring precipitation compensating summer drought effect on growth may be only apply to the earlier decades of the 21^st^ century (Fig. 2).

The model includes several simplifying assumptions that likely underestimate the potential for vegetation shifts. First, the model does not simulate drought-related cohort mortality resulting from hydraulic failure or carbon starvation. Indeed, several studies have documented large-scale tree mortality events in forests affected by drought^37–39^, including the recent (2012–2015) major drought in the Klamath region^40^; and projections suggest that the frequency of droughts will increase in this region^41^. In addition, empirical data have shown that larger trees may be more vulnerable to drought-related mortality^42^, which could further reinforce the transitions via fuel dryness enhancement and increased seedling mortality due to a decrease in canopy shading. Second, our model assumes full phenological adaptation, which may be unrealistic. Indeed, it is likely that growth may be further constrained by maladaptations to new phenological cycles^43^. Finally, the model does not incorporate the potential effects of CO_2_ fertilization^44,45^ that could accelerate forest development and growth, as well as alter successional dynamics^46,47^. The empirical effects of CO_2_ on growth in this forest are not well understood, and potential growth is likely to be limited by the low N deposition in the region (0.12 kg N/ha annual average, NADP; http://nadp.sws.uiuc.edu/).

The establishment niche has been posited as one of the key mechanisms for species to shift ranges and for the restoration of ecosystems^48^. Establishment probabilities were projected to decline under climate change due to lower soil moisture and higher temperatures (Table 1). Establishment declines have also been predicted for Mediterranean tree seedlings under drier conditions using a network of common garden experiments^18^.

Overall, the combined effect of decreased growth, decreased conifer establishment, and increased fire sizes have led to a substantial dominance shift from conifers to shrubland-hardwood in our simulations under future climate change projections. Thus, if climate change drives larger patch sizes of high-severity fire (Fig. 3d), while also creating a more arid post-fire environment, the drier portions of the landscape could face a substantial lengthening of the time to forest recovery, thereby increasing the probability that the post-fire shrubland-hardwood vegetation will be perpetuated by repeated fire, diminishing the opportunity for the system to return to forest cover^49^. Such influences appear to have played out after recent fires in the Klamath^17^, and similar decreases in potential for seedling establishment under drier conditions have been observed in other regions of the western US^18,50^, especially following high severity fires^51,52^. Our simulations, on the other hand, are not able to determine whether transitions from conifers to shrubland-hardwood are going to be persistent over the 22^nd^ century because climate change projections are temporally limited (85 yrs). This limitation makes it very difficult to assess the magnitude of such transitions, but the mechanisms modeled here (growth, establishment, and fire dynamics) showed clear indications that this could be the case.

### The current extent of conifer dominance is already in disequilibrium

Surprisingly, simulations that projected the current baseline climate (1949–2010) led to the loss of almost one-third of the initial conifer forest extent. We argue that these losses largely stem from the disequilibrium of the present vegetation conditions with current climate and disturbance dynamics.

Several lines of evidence suggest that species distributions and communities can reflect past rather than current environments^4,6,53–55^ (i.e., disequilibrium). For instance, in a climate change model experiment^56^, researchers predicted that forest species in Spain will increase their range and abundance under current climate as a result of continuing slow expansion, since many species were pushed southwards in Europe during the last glacial maximum. In the Klamath, the influence of a reduction in severe fire during the Little Ice Age, or a period of frequent but lower-severity fire in 1700–1900 may have increased the dominance of conifer forests^57,58^. That legacy may increase the vulnerability to higher fire activity today in a remarkably warmer climate (relative to 1949–2010, our baseline climate input).

In addition, decades of very effective fire suppression have promoted conifer dominance through strengthened positive fire–vegetation feedbacks, thereby facilitating extensive vegetation transformation^3,55,59,60^. Indeed, empirical data indicate that such afforestation may increase subsequent fire risk, due to increased fuel continuity and exposure to high severity fires, at least during early stages of conifer development before negative forest feedbacks with fire arise. These unintended effects, together with rapid increases in temperature, have led to an increase in fire sizes and the number of megafires in many regions^61^, as simulated here (Fig. 4).

It is challenging to parse the impacts of future climate change in forest ecosystems that are already in disequilibrium with the current climate. Such modeling experiments are more useful when interpreting the risk of such fire-induced transformation of forests, rather than trying to determine the effect of climate change on forests. Accordingly, our study shows that climate change can increase the risk of high fire activity, with broad interquartile ranges in fire rotation periods (Fig. 3) as well as peaks of large fires (Fig. 4).

Our study suggests that current conifer forests may be holding a considerable amount of resilience debt^7^ — i.e. communities are likely to be extirpated because they are not adapted to the disturbance regime —, likely to be paid during the 21^st^ century. This is likely to be the result of: (1) the current disequilibrium of conifer dominance, (2) the projected changes in the fire regime, and (3) the increasingly unfavorable conditions for conifer recovery after fire.

Further research is needed to understand to what extent local forest management can buffer against our projected forest loss, e.g. by reducing the vulnerability of conifer forests to severe fire or facilitating their post-fire recovery. Researchers will need to define when and where preventing such loss may no longer be possible.

## Methods

### Study area and species

The Klamath forest landscape is situated in the Pacific Northwest region of the United States (Fig. 1) at the convergence of major North American floristic zones, and includes an exceptionally diverse flora, with strong components of sclerophyllous broadleaf hardwood, coniferous, and herbaceous vegetation^62^. Topography is mountainous and complex; elevation ranges from 100 m to 2000 m.a.s.l. The climate is Mediterranean, with dry, warm summers and wet, mild winters. Due to the complexity of the terrain and its geographic position, a wide range of temperatures (mean annual temperature: 7–17 °C; Fig. 1) and precipitation (annual accumulated: 481–4845 mm; Fig. 1) are found in our study region. Before the onset of effective fire suppression (c. 1945), wildfire return intervals ranged from 6 to 60 years^9,57^, although the Klamath region has supported various fire regimes and return intervals in the last 2000 years^58^.

### Modeling framework

Our approach consists of modeling vegetation succession interactions with climate change to integrate both slow (e.g. forest stand development) and fast (e.g. disturbances) processes into projections of potential vegetation transitions at a regional scale^63^.

We simulated vegetation dynamics across the 2.94 million hectare Klamath region at a 270-m resolution using the forest community model LANDIS-II v 6.1^64^; http://www.landis-ii.org/). This process-based model includes biophysical (climate and soils) and ecological (species interactions, dispersal) processes to simulate growth, mortality, and regeneration at the species level. Different life history traits enable modeling inter- and intra-specific interactions (e.g. competition, facilitation), integrated with disturbance responses (e.g. resprouting, serotiny). Species were simulated as age-cohorts that compete for and modify aboveground and belowground resources within each cell (assuming homogeneity of resources and environment within a cell); disturbances and dispersal are spatially-explicit processes. The model has been widely used in both temperate forests and Mediterranean-type ecosystems to investigate climate-fire-vegetation interactions^65–67^. We ran nine model replications for the baseline and each of four climate change scenarios (see Input geophysical data section), resulting in a total of forty-five simulations (see Input geophysical data below). We classified each cell as either conifer or shrubland-hardwood state based on the functional identity (e.g. shrubland-hardwood or conifer) of the species with the highest biomass.

### Initial species distribution

Initial distribution maps of species were obtained from nearest neighbor imputation of forest inventory plots^68^. This methodology assigns each cell within the study area a forest inventory and analysis plot (FIA; United States Forest Service, USDA 2008) based on the similarity of environmental data and remote sensing image spectral properties. We aggregated cells of initial resolution of 30 m to cells of 270 m (7.20 ha), though still retained the capacity to identify species distributions at finer scales^69^. We chose to model the most prevalent target tree species and grouped shrub species into functional groups according to their seed versus resprouting behavior (see Succession section). We used the same initial distribution map for each climate scenario and climate replicate.

### Input geophysical data

Weather data were extracted from the United States Geological Service data portal (http://cida.usgs.gov/gdp/; June 2016). We used historical weather data for baseline conditions for the period 1949–2010^70^ and bias-corrected constructed analogs v2 of daily weather for climate change projections. We chose four GCM (global circulation models) – RCP (representative concentration pathways) scenarios that portray the breadth of climate change conditions predicted for the study area (ACCESS 8.5 (Ac85, hotter and drier); CanESM2 (Ca85, hotter and wetter); CNRM-CM5 4.5 (Cn45, slightly hotter and slightly wetter), MIROC5 2.6 (Mi26, slightly hotter and slightly drier; Table 1, Supplementary Fig. S1). These scenarios span the range of predicted projected changes in average annual temperature and precipitation.

Soil characteristics were obtained through the web soil survey (http://websoilsurvey.sc.egov.usda.gov/App/HomePage.htm) from the STATSGO2 database. We used the measures of soil organic matter content and physical characteristics of soils that drive water balance dynamics and biogeochemistry in the model (e.g. drainage class, soil texture). Nitrogen (N) deposition was obtained from the National Atmospheric Deposition Program database (http://nadp.sws.uiuc.edu/).

In order to harmonize different scales and data types among physical inputs, we parsed the study area into ecoregions with similar environmental conditions^71^. We grouped different cells in environmental space according to their soil and climate characteristics through cluster analysis, using the *clara* function in the ‘cluster’ package (v 2.0.4) in R 3.3.3^72^. We used soil drainage characteristics, field capacity, percentage of clay, percentage of sand, wilting point, and soil organic content to define five soil regions. We defined five climate regions using precipitation, minimum temperature, and maximum temperature. The final set of twenty-five ecoregions were derived from the combination of climate and soil regionalization sets (5 × 5).

### Succession

Vegetation succession was generated by simulating competition for light, water, and N within each cell, and is represented via C and N cycling through leaf, wood, fine and coarse roots, and by species and age cohorts using the LANDIS-II Century Extension^73^. The succession model operates at a monthly scale and simulates growth as a function of water, temperature, and available N, while accounting for inter-cohort competition for growing space. Mortality was caused by disturbances (see next section), senescence (ongoing loss of leaves and branches), and age (higher mortality rate when approaching species longevity). Regeneration and establishment were characterized by probabilities based on species-specific life history attributes that consider seed dispersal distances, sexual maturity, post-fire behavior (e.g. serotiny, resprouting), light, and water availability.

Functional groups were created by combining growth forms (e.g. hardwood, conifer, shrub), biogeochemical behavior (e.g. N-fixing), and phenology (e.g. evergreen, deciduous)^34,66^. Species and functional group parameters determined growth in response to climate and soil properties.

### Fire

Wildfire was simulated using the Dynamic Fires and Fuels extensions for the LANDIS-II model^74^. These extensions simulated the landscape-scale fire regime based on topography, fuel type, fuel condition, and daily fire weather^65,74^. Weather changed the probability of ignition and fire spread rates according to fuel type, fuel condition, and the probability of a sustained flame, via fire weather indices^75^. Fire caused mortality in tree cohorts according to their age and the species-level tolerance to fire of a given intensity. Higher fire intensity was required to kill older cohorts, whereas younger cohorts can be killed by less intense fire; fire intensity is a function of localized weather conditions and vegetation (via fuel types).

We accounted for geographic variation in fire regimes by dividing the study area into three regions reflecting differences in contemporary ignition rates and fuel moisture (Supplementary Figs S5 and S6). These regions were identified by combining remote sensing estimates of the fog belt^76^ and density estimators of fire occurrence as distance from roads and human settlements (Supplementary Fig. S5). Fuel classifications were species- and age-specific and were derived from similar ecosystems^65^.

### Model parameterization

We used literature parameters to calibrate forest succession to simulate two main processes, growth and fire effects that determine community type in the Klamath. The Century extension simulates aboveground and belowground growth of each cohort, on each site, on a monthly basis^73^. To calculate growth, the model integrates species-specific life history attributes (e.g., longevity, shade tolerance) with climate and soil conditions to estimate growth limitations imposed by competition (i.e. the biomass of other cohorts relative to the amount of maximum potential biomass) and abiotic conditions, like water availability, N availability and temperature. The parameters we selected to calibrate growth were: (1) maximum monthly aboveground productivity, and (2) large wood mass [g C/m^2^] at which half of the theoretical maximum leaf area is attained. We compared model output to 950 Forest Inventory Analysis (FIA) plots representative of the different community types and climate gradients present in the study area. Accuracy assessment indicated that the model was able to capture species-specific biomass with an average deviation of 10% of the biomass in a plot for each species (Supplementary Fig. S3). A complete list of input parameters is available in Supplementary Tables S4–S14.

We parameterized the fire regime based on the fire rotation period, fire size distribution, and fire severity^77^. These parameters were derived using data available from the Monitoring Trends in Burned Severity program^78^, which is based on pre- and post-fire LANDSAT imagery at 30-m resolution. We chose the period 2000–2010 as fire calibration period. For the fire size calibration, we excluded the 2002 Biscuit Fire (ca. 202,000 ha), because we wanted to discount the effect of a single fire that had a large influence over the fire regime (Supplementary Fig. S4 and Supplementary Table S15). Fire severity derived from the Difference Normalized Burned Ratio Index was converted to percent crown mortality using equations developed in the study area^79^. Fire size, severity, and frequency were accurately reproduced by the model (Supplementary Table S16; input parameters in Supplementary Tables S17 and S18). The median fire size differed by 29 ha (1,206 ha in the MTBS empirical data, 1,177 in the simulated period). The 90^th^ percentile of fire sizes differed by 797 ha (11,888 ha and 11,091 ha). Fire rotation period differed by only 8 years (120 yr. in the MTBS empirical data, 122 yr. for model simulation), and fire severity – converted to percentage of crown damage – differed by 12% (55% for the calibration period, whereas 68% for the model simulation).

### Data availability

All the data required to run the model are freely available online and specified in the methods sections. Specific model parameters are included in Supplementary Information.

## Electronic supplementary material

Supplementary Information
