# Supplementary material for: Disequilibrium of fire-prone forests sets the stage for a rapid decline in conifer dominance during the 21st century
Source: Sci Rep. 2018 Apr 30;8:6749. doi: 10.1038/s41598-018-24642-2 (PMC5928035; doi:10.1038/s41598-018-24642-2)
Supplement: Supplementary file 1 — Supplementary Information [file 41598_2018_24642_MOESM1_ESM.docx]

**SUPPLEMENTARY INFORMATION**

**Disequilibrium of fire-prone forests sets the stage for a rapid decline in conifer dominance during the 21^st^ century.**

Josep M. Serra-Diaz*^1,2^, Charles Maxwell^3^, Melissa S. Lucash^4^, Robert M. Scheller^3^, Danelle M. Laflower^1^, Adam D. Miller^5^, Alan J. Tepley^5^, Howard E. Epstein^6^, Kristina J. Anderson-Teixeira^5,7^, Jonathan R. Thompson^1^.

* Corresponding author

^1^ Harvard Forest, Harvard University, Petersham, MA, USA.

^2^ Section of Ecoinformatics and Biodiversity, Department of Bioscience, Aarhus University, Ny Munkgade 116, 8000 Aarhus C, Denmark.

^3^ Department of Environmental Science and Management, Portland State University, P.O. Box 751, Portland, OR 97207, USA

^4^ Department of Geography, Portland State University, P.O. Box 751, Portland, OR 97207, USA

^5^ Conservation Ecology Center, Smithsonian Conservation Biology Institute; Front Royal, Virginia, USA.

^6^ Department of Environmental Sciences, University of Virginia, Charlottesville, VA, US.

^7^ Center for Tropical Forest Science, Smithsonian Tropical Research Institute; Panama.

Table S1. Test for significant differences in Fire Rotation Periods between climate change scenarios. Shapiro test for normality and subsequent Dunnett’s test for multiple group comparison with the control (Historical simulations).

1 Shapiro test results

| scenario | W | p |
| --- | --- | --- |
| ACCESS_rcp85 | 0.935379 | 0.534186 |
| CanESM2_rcp85 | 0.940686 | 0.589142 |
| CNRMCM5_rcp45 | 0.859294 | 0.094202 |
| HIST | 0.96801 | 0.877194 |
| MIROC5_rcp26 | 0.890685 | 0.202787 |

2 Dunnett’s test for multiple comparisons

|  | Estimate | Std. Errr | t value | Pr(>\|t\|) |
| --- | --- | --- | --- | --- |
| MIROC5_rcp26 - HIST | -2.797 | 7.222 | -0.387 | 0.9856 |
| CNRMCM5_rcp45 - HIST | -7.91 | 7.222 | -1.095 | 0.647 |
| CanESM2_rcp85 - HIST | -17.153 | 7.222 | -2.375 | 0.0731 |
| ACCESS_rcp85 - HIST | -19.336 | 7.222 | -2.677 | **0.0364** |

Table S2. Test for significant differences in mean fire size between climate change scenarios. Shapiro test for normality and subsequent Dunnett’s test for multiple group comparison with the control (Historical simulations).

1 Shapiro test results

| scenario | W | p |
| --- | --- | --- |
| ACCESS_rcp85 | 0.957506 | 0.771889 |
| CanESM2_rcp85 | 0.952513 | 0.717646 |
| CNRMCM5_rcp45 | 0.85896 | 0.093417 |
| HIST | 0.903999 | 0.2761 |
| MIROC5_rcp26 | 0.865262 | 0.109338 |

2 Dunnett’s test for multiple comparisons

|  | Estimate | Std. Error | t value | Pr(>\|t\|) |
| --- | --- | --- | --- | --- |
| MIROC5_rcp26 - HIST | 27.73 | 448.55 | 0.062 | 1 |
| CNRMCM5_rcp45 - HIST | 501.35 | 448.55 | 1.118 | 0.6313 |
| CanESM2_rcp85 - HIST | 1004.99 | 448.55 | 2.241 | 0.0979 |
| ACCESS_rcp85 - HIST | 857.83 | 448.55 | 1.912 | 0.1885 |

Table S3. Test for significant differences in patch area of high severity fires climate change scenarios. Shapiro test for normality and subsequent Dunnett’s test for multiple group comparison with the control (Historical simulations).

1Test of differences among groups

| scenario | W | p |
| --- | --- | --- |
| Ac85 | 0.964271 | 0.849654 |
| Base | 0.913949 | 0.382711 |
| Ca85 | 0.831518 | 0.061563 |
| Cn45 | 0.901597 | 0.298653 |
| Mi26 | 0.873706 | 0.163791 |

2 Dunnett’s test for multiple comparisons

|  | Estimate | Std. Error | t -value | Pr(>\|t\|) |
| --- | --- | --- | --- | --- |
| MIROC5_rcp26 - HIST | 7418 | 12133 | 0.611 | 0.92904 |
| CNRMCM5_rcp45 - HIST | 4048 | 12133 | 0.334 | 0.99165 |
| CanESM2_rcp85 - HIST | 57515 | 12133 | 4.741 | **0.00021** |
| ACCESS_rcp85 - HIST | 44649 | 12133 | 3.68 | **0.00295** |

Figure S1. Climate change scenarios considered.

#
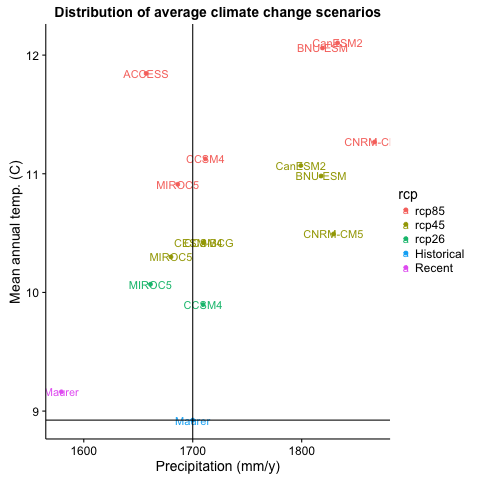


Figure S2. Species-level biomass calibration. All simulated medians were within the range of 40 to 60 percentile in the expected values. Species code key: PSME (*Pseduotsuga menziesii* - Conifer); PIMO3 (*Pinus monticola* - Conifer)*;* CADE27 (*Calocedrus decurrens* - Conifer); ABPRSH ( *Abies procera* and *Abies magnifica var. shastensis*- Conifer)*;* PILA (*Pinus lambertiana*- Conifer)*;* ABGRC (*Abies grandis* / *A. concolor*- Conifer); PIPO (*Pinus ponderosa*- Conifer)*;* ARME (*Arbutus menziesii* - Hardwood)*;* CHCH7 (*Chrysolepsis chrysophylla* - Hardwood); QUGA4 (*Quercus garryana* - Hardwood); LIDE3 (*Lithocarpus densiflors* - Hardwood)*;* QUKE (*Quercus keloggii* - Hardwood)*;* QUCH2 (*Quercus chrysolepsis* - Hardwood).


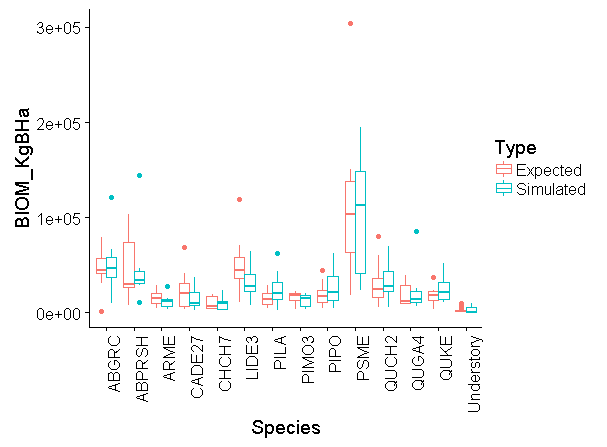


Figure S3. Plot level accuracy assessment. Color indicates ecoregion, dot size indicates the community importance (frequency) within the study area.

No observable systematic bias was seen across ecoregions. There was a slight underestimation of very high biomass plots. There was no bias by ecoregion.


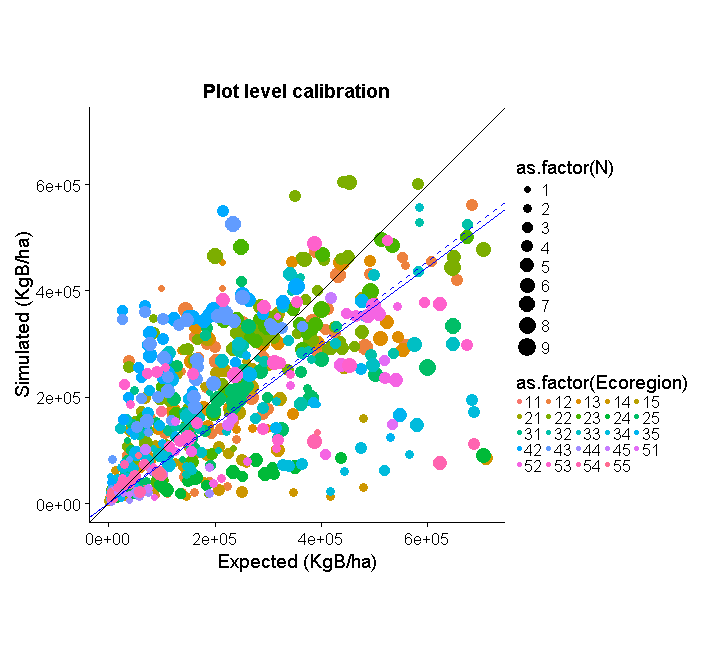


Figure S4. Total burned area for the period 1984-2010, estimated from the Monitoring Trends in Burned Severity Program (MTBS; (Eidenshink *et al.*, 2007).


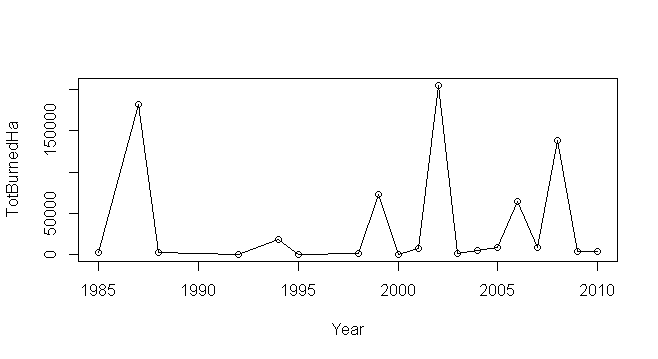


Figure S5. Cumulative ratio of fire ignitions from distance to road estimated cause of ignition using the Federal Ignitions dataset (Short, 2013). *y* is the ratio of ignitions over the total and *NEAR_DIST (m)*  represents the minimum distance, in meters, from the fire to a road/human settlement. A threshold of 1500 m was chosen to design different fire regions, since it is the distance at which natural and human caused ignitions differentiate the most.
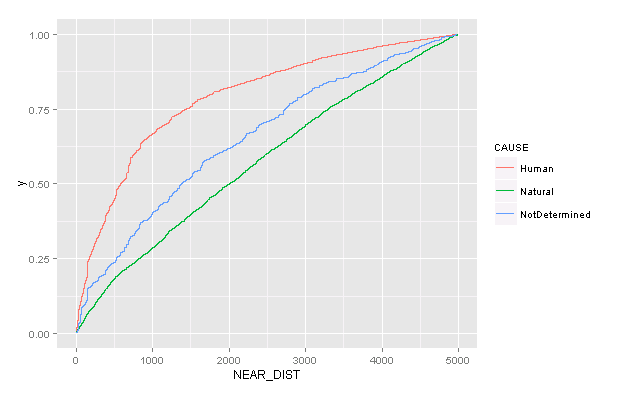


Figure S6. Fire regions encompassing spatial variability in ignitions and spread rates. Fire region 1 (41% of ignitions) characterizes forested area with dominance of natural fire ignitions (>1500m from roads, see Fig. S4). Fire region 2 characterizes fire in forest with high influence of human ignitions (54% of ignitions; <1500 m. from roads, see Fig. S4) and Fire region 3 characterizes a lower number of ignitions and fire activity (5% of ignitions). We used the information on the USGS (Torregrosa *et al.*, 2016) for decadal hours of fog. Subsequently, we identified the threshold of fog in fires captured by the Monitoring Trends in Burned Severity Program (MTBS; (Eidenshink *et al.*, 2007). We used the MTBS dataset to assess when a large fire spread in the fog region and used that to delimit fogs, together from a distance threshold from the coastline. *Maps were created using ArcGIS v.10.5 (www.ersi.com/argis).*


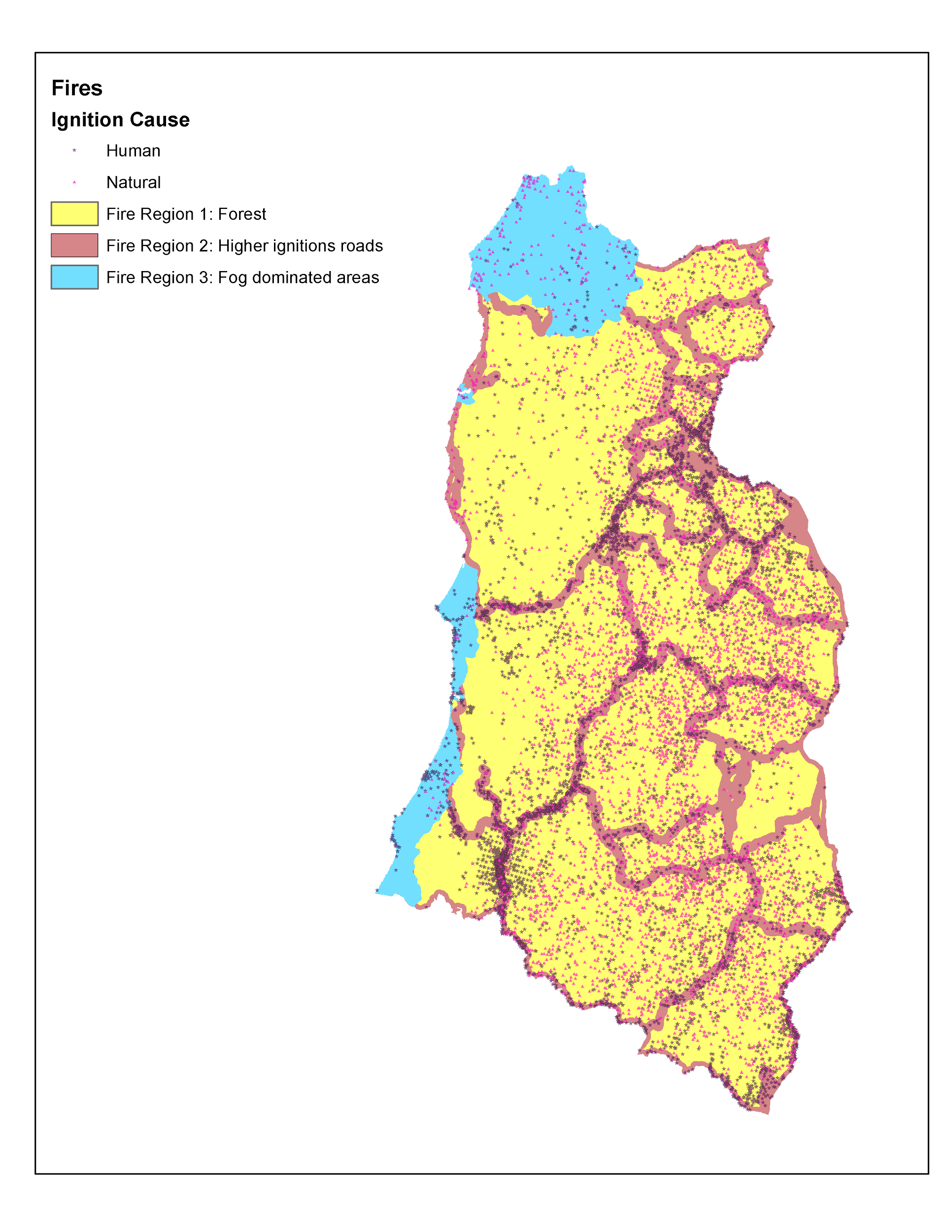

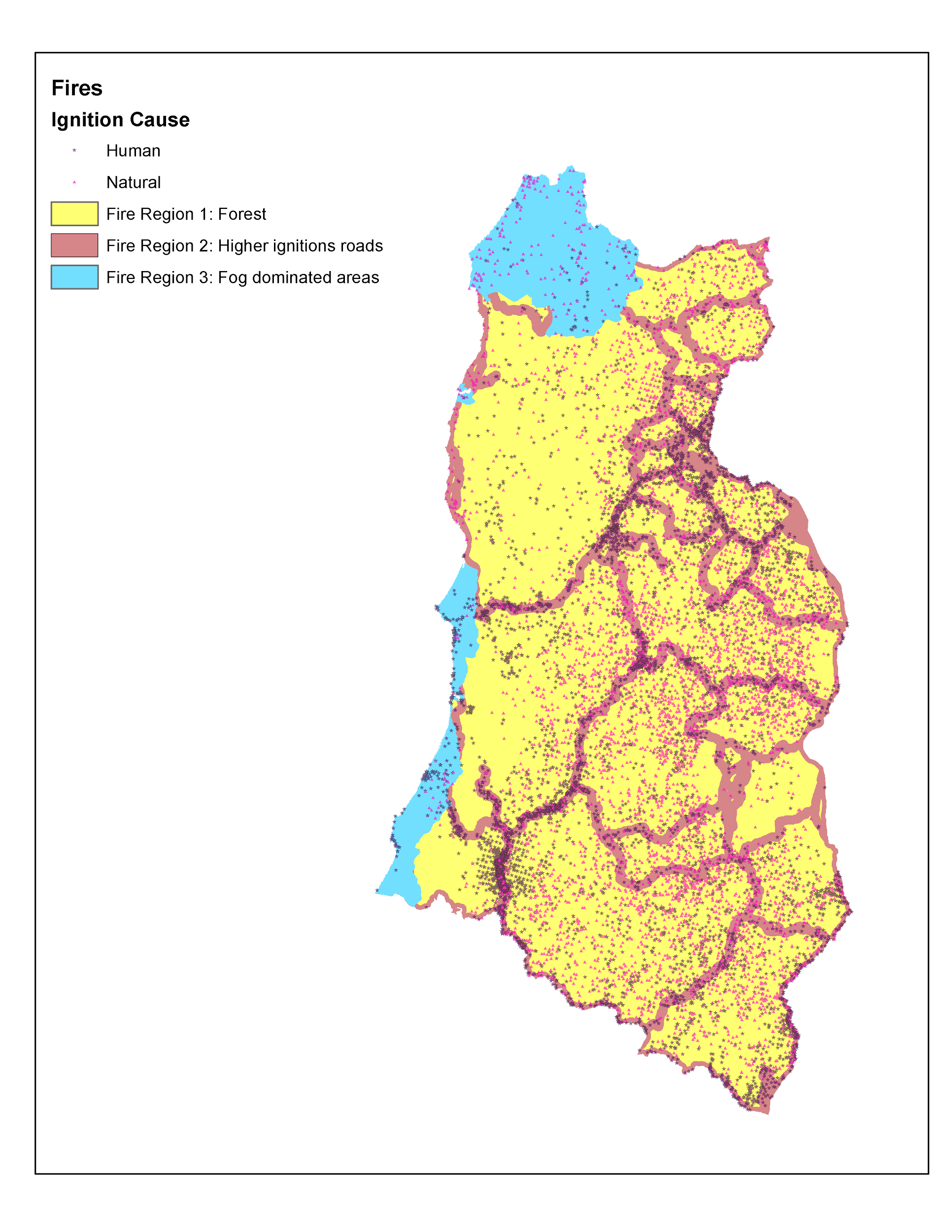


Figure S7. Mean fire rotation thresholds on predictions of conifer dominance in forests for the period 2070-2100. Plot is a conditional inference classification tree using the Party Package v1.2-1 in R.


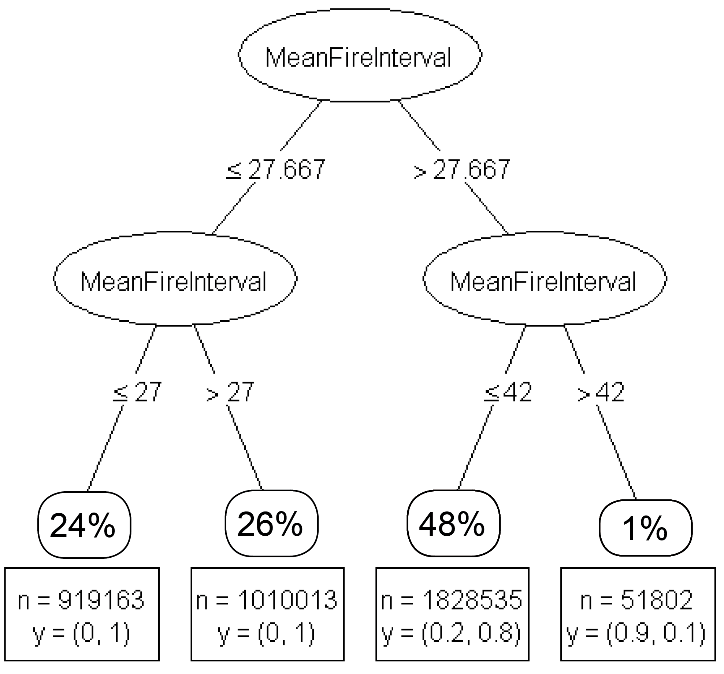


**TABLES**

Table S4. LANDIS-II general species parameters.

| Species | Longe-vity ^1-6^ | Sexual maturity ^1-3, 5, 7^ | Shade tolerance ^1, 3^ | Fire tolerance ^2^ | Seed dispersal distance | | Vegetative reproduction probability ^1-2, 5^ | Sprout age | | Post-fire regener-ation ^1-2^ |
| --- | --- | --- | --- | --- | --- | --- | --- | --- | --- | --- |
|  |  |  |  |  | Effective ^1-3, 8-9^ | Max  ^1-3, 9^ |  | Min ^2,5^ | Max ^1-2^ |  |
| ABGRC | 500 | 35 | 3 | 3 | 30 | 500 | 0 | 0 | 0 | none |
| APBRSH | 650 | 40 | 3 | 3 | 30 | 500 | 0 | 0 | 0 | none |
| ARME | 350 | 5 | 3 | 2 | 200 | 800 | 0.8 | 4 | 300 | resprout |
| CADE27 | 650 | 30 | 3 | 5 | 30 | 2000 | 0 | 0 | 0 | none |
| CHCH7 | 250 | 40 | 3 | 3 | 100 | 800 | 0.8 | 2 | 200 | resprout |
| FX_Resp_Deciduous | 250 | 2 | 2 | 1 | 30 | 500 | 0.75 | 5 | 200 | resprout |
| FX_Seed_Deciduous | 250 | 2 | 2 | 1 | 30 | 800 | 0 | 0 | 200 | none |
| FX_Seed_Evergreen | 250 | 2 | 2 | 1 | 30 | 800 | 0 | 0 | 200 | none |
| LIDE3 | 250 | 3 | 4 | 3 | 100 | 400 | 0.8 | 2 | 200 | resprout |
| NoFX_Resp_Deciduous | 250 | 2 | 2 | 1 | 30 | 550 | 0.85 | 5 | 200 | resprout |
| NoFX_Resp_Evergreen | 250 | 2 | 2 | 1 | 30 | 550 | 0.85 | 5 | 200 | resprout |
| NoFX_Seed_Deciduous | 250 | 2 | 2 | 1 | 30 | 1000 | 0 | 0 | 200 | none |
| NoFX_Seed_Evergreen | 250 | 2 | 2 | 1 | 30 | 1000 | 0 | 0 | 200 | none |
| PILA | 750 | 20 | 3 | 4 | 30 | 400 | 0 | 0 | 700 | none |
| PIMO3 | 600 | 18 | 3 | 4 | 30 | 800 | 0 | 0 | 550 | none |
| PIPO | 850 | 25 | 4 | 4 | 50 | 300 | 0 | 0 | 800 | none |
| PSME | 650 | 15 | 3 | 3 | 140 | 2000 | 0 | 0 | 600 | none |
| QUCH2 | 250 | 20 | 3 | 2 | 30 | 1000 | 0.95 | 1 | 200 | resprout |
| QUGA4 | 350 | 40 | 2 | 2 | 100 | 400 | 0.8 | 3 | 300 | resprout |
| QUKE | 300 | 30 | 3 | 2 | 100 | 400 | 0.9 | 3 | 250 | resprout |

^1^ Burns & Honkala 1990

^2^ Innes 2013

^3^ Tree Species Compendium

^4^ Dale *et al.,* 1986

^5^ Niemiec *et al.,* 1995

^6^ Franklin & Waring 1980

^7^ Harrington *et al.,* 2006

^8^ Beach & Halpern 2001

^9^ Bonner & Karrfalt 2008

Table S5. Available light biomass table.

| Shade class | All ecoregions |
| --- | --- |
| 1 | 10% |
| 2 | 20% |
| 3 | 40% |
| 4 | 65% |
| 5 | 95% |

Table S6. Light establishment table.

| Species shade class | Probability by actual shade | | | | | |
| --- | --- | --- | --- | --- | --- | --- |
|  | 0 | 1 | 2 | 3 | 4 | 5 |
| 1 | 1.0 | 0.5 | 0.02 | 0.0 | 0.0 | 0.0 |
| 2 | 0.6 | 1.0 | 0.5 | 0.02 | 0.0 | 0.0 |
| 3 | 0.01 | 0.6 | 1.0 | 0.5 | 0.02 | 0.0 |
| 4 | 0.0 | 0.01 | 0.6 | 1.0 | 0.5 | 0.02 |
| 5 | 0.0 | 0.0 | 0.01 | 0.5 | 1.0 | 1.0 |

Table S7. Century succession species parameters. GDD: growing degree days, N: nitrogen, C: carbon.

| Species | Functional Type | N Fixer | GDD Min ^1^ | GDD Max ^1^ | Min January Temp ^1^ | Drought Tolerance | Leaf Longevity ^2-4^ | Epicormic Resprout ^5^ |
| --- | --- | --- | --- | --- | --- | --- | --- | --- |
|  |  |  |  |  |  |  |  |  |
| PSME | 2 | N | 300 | 4750 | -12 | 0.7 | 6 | N |
| PIMO3 | 1 | N | 155 | 1220 | -18 | 0.82 | 7 | N |
| CADE27 | 2 | N | 1150 | 3550 | -2 | 0.7 | 5 | N |
| ABPRSH | 6 | N | 550 | 1900 | -5 | 0.5 | 5 | N |
| PILA | 2 | N | 1050 | 3300 | -2 | 0.7 | 5 | N |
| ABGRC | 7 | N | 500 | 2450 | -9 | 0.7 | 6 | N |
| PIPO | 2 | N | 500 | 5500 | -9 | 0.9 | 5 | N |
| ARME | 4 | N | 900 | 3700 | 1 | 0.8 | 1 | N |
| CHCH7 | 4 | N | 950 | 2950 | -1 | 0.7 | 3 | N |
| QUGA4 | 4 | N | 900 | 3050 | 0 | 0.9 | 1 | Y |
| LIDE3 | 3 | N | 1250 | 3150 | 2 | 0.9 | 1 | Y |
| QUKE | 4 | N | 1250 | 3850 | -1 | 0.9 | 1 | Y |
| QUCH2 | 4 | N | 900 | 3050 | 0 | 0.9 | 1 | Y |
| FX_Resp_Deciduous | 5 | Y | 300 | 5000 | -10 | 0.99 | 1 | N |
| NoFX_Resp_Deciduous | 5 | N | 300 | 5000 | -10 | 0.97 | 1 | N |
| FX_Seed_Deciduous | 5 | Y | 300 | 5000 | -10 | 0.99 | 1 | Y |
| NoFX_Seed_Deciduous | 5 | N | 300 | 5000 | -10 | 0.97 | 1 | N |
| NoFX_Resp_Evergreen | 5 | N | 300 | 5000 | -10 | 0.97 | 5 | N |
| FX_Seed_Evergreen | 5 | Y | 300 | 5000 | -10 | 0.99 | 5 | N |
| NoFX_Seed_Evergreen | 5 | N | 300 | 5000 | -10 | 0.97 | 5 | N |

Table S8, continued.

| Species | Lignin Content ^6-12^ | | | | C:N Ratio ^6-8, 11, 13-27^ | | | | |
| --- | --- | --- | --- | --- | --- | --- | --- | --- | --- |
|  | Leaf | Fine Root | Wood | Coarse Root | Leaf | Fine Root | Wood | Coarse Root | Litter |
| PSME | 0.24 | 0.3 | 0.28 | 0.32 | 42 | 36 | 400 | 80 | 77 |
| PIMO3 | 0.31 | 0.2 | 0.25 | 0.25 | 37 | 37 | 500 | 80 | 100 |
| CADE27 | 0.24 | 0.26 | 0.28 | 0.32 | 50 | 48 | 400 | 80 | 80 |
| ABPRSH | 0.24 | 0.22 | 0.28 | 0.32 | 50 | 26 | 400 | 80 | 80 |
| PILA | 0.24 | 0.25 | 0.28 | 0.32 | 50 | 38 | 400 | 80 | 75 |
| ABGRC | 0.25 | 0.22 | 0.28 | 0.32 | 42 | 27 | 400 | 80 | 77 |
| PIPO | 0.24 | 0.23 | 0.28 | 0.32 | 50 | 47 | 400 | 80 | 75 |
| ARME | 0.18 | 0.22 | 0.28 | 0.32 | 25 | 30 | 400 | 80 | 100 |
| CHCH7 | 0.18 | 0.22 | 0.28 | 0.32 | 25 | 30 | 400 | 80 | 100 |
| QUGA4 | 0.18 | 0.22 | 0.28 | 0.32 | 27 | 30 | 400 | 80 | 33 |
| LIDE3 | 0.18 | 0.22 | 0.28 | 0.32 | 27 | 30 | 400 | 80 | 33 |
| QUKE | 0.18 | 0.22 | 0.28 | 0.32 | 27 | 30 | 400 | 80 | 33 |
| QUCH2 | 0.18 | 0.22 | 0.28 | 0.32 | 27 | 30 | 400 | 80 | 33 |
| FX_Resp_Deciduous | 0.25 | 0.2 | 0.25 | 0.25 | 20 | 30 | 80 | 222 | 50 |
| NoFX_Resp_Deciduous | 0.25 | 0.2 | 0.25 | 0.25 | 59 | 59 | 80 | 222 | 100 |
| FX_Seed_Deciduous | 0.25 | 0.2 | 0.25 | 0.25 | 20 | 28 | 80 | 222 | 50 |
| NoFX_Seed_Deciduous | 0.25 | 0.2 | 0.25 | 0.25 | 59 | 59 | 80 | 222 | 100 |
| NoFX_Resp_Evergreen | 0.25 | 0.2 | 0.25 | 0.25 | 59 | 59 | 80 | 222 | 100 |
| FX_Seed_Evergreen | 0.25 | 0.2 | 0.25 | 0.25 | 59 | 59 | 80 | 222 | 100 |
| NoFX_Seed_Evergreen | 0.25 | 0.2 | 0.25 | 0.25 | 59 | 59 | 80 | 222 | 100 |

^1^ Thompson et al 2000a,b

^2^ Balster & Marshall 2000

^3^ Harlow *et al.,* 2005

^4^ Hudiburg *et al.,* 2013

^5^ Innes 2013

^6^ NERC 2013

^7^ Chen *et al.,* 2001

^8^ Chen *et al.,* 2002

^9^ Entry *et al.,* 1992

^10^ Lewis 1950

^11^ Scheller *et al.,* 2011

^12^ Schowalter & Morrell 2002

^13^ Cross & Perakis 2011

^14^ Debell & Radwan 1984

^15^ Edmonds 1980

^16^ Hobbie *et al.,* 2006

^17^ Keenan *et al.,* 1996

^19^ Pardo *et al.,* 2005

^20^ Perakis *et al.,* 2006

^21^ Pierce *et al.,* 1994

^22^ Prescott *et al.,* 2000

^23^ Prescott & Preston 1994

^24^ Scott *et al*., 2008

^25^ Thomas & Prescott 2000

^26^ Valachovic *et al*., 2004

^27^ Yang *et al*., 2010

Table S9. Century succession functional group parameters. Parameter acronyms are from the Century model (Parton *et al.,* 1983).

| Functional Group | Index | Temperature Parameters ^1-4^ | | | | Leaf Fraction ^5-9^ | Leaf Area Index Parameters ^10^ | | |
| --- | --- | --- | --- | --- | --- | --- | --- | --- | --- |
|  |  | Mean (PPDF1) | Max (PPDF2) | PPDF3 | PPDF4 |  | BTOLAI | KLAI | MaxLAI |
| Conifers_mesic | 1 | 20 | 40 | 1.1 | 6 | 0.3 | -0.8 | 15000 | 15 |
| Conifers_dry | 2 | 20 | 40 | 1.1 | 6 | 0.3 | -0.8 | 15000 | 10 |
| Hardwood_mesic | 3 | 21 | 40 | 0.8 | 6 | 0.32 | -0.8 | 17000 | 10 |
| Hardwood_dry | 4 | 21 | 40 | 0.8 | 6 | 0.32 | -0.8 | 17000 | 8 |
| Shrub | 5 | 18.5 | 55 | 2 | 8 | 0.3 | -0.8 | 4000 | 5 |
| Abies_mesic | 6 | 20 | 40 | 1.1 | 6 | 0.3 | -0.8 | 15000 | 15 |
| Abies_dry | 7 | 20 | 40 | 1.1 | 6 | 0.3 | -0.8 | 15000 | 15 |
| Hardwood_dry2 | 8 | 22.5 | 40 | 0.8 | 6 | 0.32 | -0.8 | 16000 | 8 |

Table S10, continued.

| Functional Group | Drought Parameters | | Woody Decay Rate | Monthly Wood Mortality ^6^ | Age Mortality Shape | Leaf Drop Month |
| --- | --- | --- | --- | --- | --- | --- |
|  | PPRPTS2 | PPRPTS3 |  |  |  |  |
| Conifers_mesic | 0.7 | 0.6 | 0.08 | 0.001 | 15 | 9 |
| Conifers_dry | 0.6 | 0.6 | 0.08 | 0.001 | 15 | 9 |
| Hardwood_mesic | 1.6 | 0.9 | 0.08 | 0.001 | 15 | 9 |
| Hardwood_dry | 0.1 | 0.8 | 0.08 | 0.001 | 15 | 9 |
| Shrub | 0 | 0.1 | 0.08 | 0.001 | 15 | 9 |
| Abies_mesic | 0.9 | 0.8 | 0.08 | 0.001 | 15 | 9 |
| Abies_dry | 0.9 | 0.8 | 0.08 | 0.001 | 15 | 9 |
| Hardwood_dry2 | 0.1 | 0.8 | 0.08 | 0.001 | 15 | 9 |

^1^ Burns & Honkala 1990

^2^ Innes 2013

^3^ Tree Species Compendium

^4^ Dale *et al*., 1986

^5^ Niemiec *et al*., 1995

^6^ Franklin & Waring 1980

^7^ Harrington *et al*., 2006

^8^ Beach & Halpern 2001

^9^ Lewis 1985

^10^ Law *et al.,* 2004

Table S11. Initial ecoregion parameters. SOM: soil organic matter, C: carbon, N: nitrogen. All data from STATSGO database in the study area.

|  | SOM1 C Surface | SOM1 N Surface | SOM1 C Soil | SOM1 N Soil | SOM2 C | SOM2 N | SOM3 C | SOM3 N | Mineral N |
| --- | --- | --- | --- | --- | --- | --- | --- | --- | --- |
| eco11 | 75 | 8 | 150 | 15 | 4434 | 253 | 2856 | 336 | 6 |
| eco12 | 36 | 4 | 72 | 7 | 2122 | 121 | 1367 | 161 | 3 |
| eco13 | 27 | 3 | 54 | 5 | 1583 | 90 | 1020 | 120 | 2.1 |
| eco14 | 3 | 1 | 6 | 1 | 168 | 10 | 108 | 12 | 1 |
| eco15 | 16 | 2 | 31 | 3 | 921 | 53 | 593 | 70 | 1.3 |
| eco21 | 75 | 8 | 150 | 15 | 4434 | 253 | 2856 | 336 | 6 |
| eco22 | 36 | 4 | 72 | 7 | 2122 | 121 | 1367 | 161 | 3 |
| eco23 | 27 | 3 | 54 | 5 | 1583 | 90 | 1020 | 120 | 2.1 |
| eco24 | 3 | 1 | 6 | 1 | 168 | 10 | 108 | 12 | 1 |
| eco25 | 16 | 2 | 31 | 3 | 921 | 53 | 593 | 70 | 1.3 |
| eco31 | 75 | 8 | 150 | 15 | 4434 | 253 | 2856 | 336 | 6 |
| eco32 | 36 | 4 | 72 | 7 | 2122 | 121 | 1367 | 161 | 3 |
| eco33 | 27 | 3 | 54 | 5 | 1583 | 90 | 1020 | 120 | 2.1 |
| eco34 | 3 | 1 | 6 | 1 | 168 | 10 | 108 | 12 | 1 |
| eco35 | 16 | 2 | 31 | 3 | 921 | 53 | 593 | 70 | 1.3 |
| eco42 | 36 | 4 | 72 | 7 | 2122 | 121 | 1367 | 161 | 3 |
| eco43 | 27 | 3 | 54 | 5 | 1583 | 90 | 1020 | 120 | 2.1 |
| eco44 | 3 | 1 | 6 | 1 | 168 | 10 | 108 | 12 | 1 |
| eco45 | 16 | 2 | 31 | 3 | 921 | 53 | 593 | 70 | 1.3 |
| eco51 | 75 | 8 | 150 | 15 | 4434 | 253 | 2856 | 336 | 6 |
| eco52 | 36 | 4 | 72 | 7 | 2122 | 121 | 1367 | 161 | 3 |
| eco53 | 27 | 3 | 54 | 5 | 1583 | 90 | 1020 | 120 | 2.1 |
| eco54 | 3 | 1 | 6 | 1 | 168 | 10 | 108 | 12 | 1 |
| eco55 | 16 | 2 | 31 | 3 | 921 | 53 | 593 | 70 | 1.3 |

Table S12. Ecoregion parameter table. Soil organic matter (SOM) is divided into four pools (SOM1-surface, SOM1-soil, SOM2 and SOM3) based on the Century soil model (Parton *et al.,* 1983).

| Ecoregion | Soil Depth (cm) | Clay Content ^1^ | Sand Content ^1^ | Field Capacity ^1^ | Wilting Point ^1^ | Storm Flow Fraction | Base Flow Fraction | Drainage Class ^1^ |
| --- | --- | --- | --- | --- | --- | --- | --- | --- |
|  |  |  |  |  |  |  |  |  |
| eco11 | 100 | 0.26 | 0.27 | 0.27 | 0.15 | 0.2 | 0.2 | 1 |
| eco12 | 100 | 0.23 | 0.42 | 0.18 | 0.09 | 0.2 | 0.2 | 1 |
| eco13 | 100 | 0.24 | 0.42 | 0.2 | 0.11 | 0.2 | 0.2 | 1 |
| eco14 | 100 | 0.19 | 0.51 | 0.03 | 0.02 | 0.2 | 0.2 | 1 |
| eco15 | 100 | 0.22 | 0.46 | 0.09 | 0.05 | 0.2 | 0.2 | 1 |
| eco21 | 100 | 0.26 | 0.27 | 0.27 | 0.15 | 0.2 | 0.2 | 1 |
| eco22 | 100 | 0.23 | 0.42 | 0.18 | 0.09 | 0.2 | 0.2 | 1 |
| eco23 | 100 | 0.24 | 0.42 | 0.2 | 0.11 | 0.2 | 0.2 | 1 |
| eco24 | 100 | 0.19 | 0.51 | 0.03 | 0.02 | 0.2 | 0.2 | 1 |
| eco25 | 100 | 0.22 | 0.46 | 0.09 | 0.05 | 0.2 | 0.2 | 1 |
| eco31 | 100 | 0.26 | 0.27 | 0.27 | 0.15 | 0.2 | 0.2 | 1 |
| eco32 | 100 | 0.23 | 0.42 | 0.18 | 0.09 | 0.2 | 0.2 | 1 |
| eco33 | 100 | 0.24 | 0.42 | 0.2 | 0.11 | 0.2 | 0.2 | 1 |
| eco34 | 100 | 0.19 | 0.51 | 0.03 | 0.02 | 0.2 | 0.2 | 1 |
| eco35 | 100 | 0.22 | 0.46 | 0.09 | 0.05 | 0.2 | 0.2 | 1 |
| eco42 | 100 | 0.26 | 0.27 | 0.27 | 0.15 | 0.2 | 0.2 | 1 |
| eco43 | 100 | 0.24 | 0.42 | 0.2 | 0.11 | 0.2 | 0.2 | 1 |
| eco44 | 100 | 0.19 | 0.51 | 0.03 | 0.02 | 0.2 | 0.2 | 1 |
| eco45 | 100 | 0.22 | 0.46 | 0.09 | 0.05 | 0.2 | 0.2 | 1 |
| eco51 | 100 | 0.26 | 0.27 | 0.27 | 0.15 | 0.2 | 0.2 | 1 |
| eco52 | 100 | 0.23 | 0.42 | 0.18 | 0.09 | 0.2 | 0.2 | 1 |
| eco53 | 100 | 0.24 | 0.42 | 0.2 | 0.11 | 0.2 | 0.2 | 1 |
| eco54 | 100 | 0.19 | 0.51 | 0.03 | 0.02 | 0.2 | 0.2 | 1 |
| eco55 | 100 | 0.22 | 0.46 | 0.09 | 0.05 | 0.2 | 0.2 | 1 |

Table S12, continued.

| Ecoregion | Nitrogen Inputs ^2-5^ | | Lati-tude | SOM Decay Rates | | | | Denitri-fication |
| --- | --- | --- | --- | --- | --- | --- | --- | --- |
|  | Intercept | Slope |  | SOM1 surface | SOM1 soil | SOM2 | SOM3 |  |
| eco11 | 0.001 | 0.2 | 41 | 0.4 | 0.35 | 0.1 | 0.0003 | 0 |
| eco12 | 0.001 | 0.2 | 41 | 0.4 | 0.35 | 0.1 | 0.0003 | 0 |
| eco13 | 0.001 | 0.2 | 41 | 0.4 | 0.35 | 0.1 | 0.0003 | 0 |
| eco14 | 0.001 | 0.2 | 41 | 0.4 | 0.35 | 0.1 | 0.0003 | 0 |
| eco15 | 0.001 | 0.2 | 41 | 0.4 | 0.35 | 0.1 | 0.0003 | 0 |
| eco21 | 0.001 | 0.2 | 41 | 0.4 | 0.35 | 0.1 | 0.0003 | 0 |
| eco22 | 0.001 | 0.2 | 41 | 0.4 | 0.35 | 0.1 | 0.0003 | 0 |
| eco23 | 0.001 | 0.2 | 41 | 0.4 | 0.35 | 0.1 | 0.0003 | 0 |
| eco24 | 0.001 | 0.2 | 41 | 0.4 | 0.35 | 0.1 | 0.0003 | 0 |
| eco25 | 0.001 | 0.2 | 41 | 0.4 | 0.35 | 0.1 | 0.0003 | 0 |
| eco31 | 0.001 | 0.2 | 41 | 0.4 | 0.35 | 0.1 | 0.0003 | 0 |
| eco32 | 0.001 | 0.2 | 41 | 0.4 | 0.35 | 0.1 | 0.0003 | 0 |
| eco33 | 0.001 | 0.2 | 41 | 0.4 | 0.35 | 0.1 | 0.0003 | 0 |
| eco34 | 0.001 | 0.2 | 41 | 0.4 | 0.35 | 0.1 | 0.0003 | 0 |
| eco35 | 0.001 | 0.2 | 41 | 0.4 | 0.35 | 0.1 | 0.0003 | 0 |
| eco42 | 0.001 | 0.2 | 41 | 0.4 | 0.35 | 0.1 | 0.0003 | 0 |
| eco43 | 0.001 | 0.2 | 41 | 0.4 | 0.35 | 0.1 | 0.0003 | 0 |
| eco44 | 0.001 | 0.2 | 41 | 0.4 | 0.35 | 0.1 | 0.0003 | 0 |
| eco45 | 0.001 | 0.2 | 41 | 0.4 | 0.35 | 0.1 | 0.0003 | 0 |
| eco51 | 0.001 | 0.2 | 41 | 0.4 | 0.35 | 0.1 | 0.0003 | 0 |
| eco52 | 0.001 | 0.2 | 41 | 0.4 | 0.35 | 0.1 | 0.0003 | 0 |
| eco53 | 0.001 | 0.2 | 41 | 0.4 | 0.35 | 0.1 | 0.0003 | 0 |
| eco54 | 0.001 | 0.2 | 41 | 0.4 | 0.35 | 0.1 | 0.0003 | 0 |
| eco55 | 0.001 | 0.2 | 41 | 0.4 | 0.35 | 0.1 | 0.0003 | 0 |

^1^ Soil Survey Staff, Natural Resources Conservation Service

^2^ Fenn *et al.,* 2003

^3^ Johnson *et al.,* 1982

^4^ Sollins *et al*., 1980

^5^ Zhang *et al.,* 2012

Table S13. Monthly maximum above-ground net primary productivity (ANPP) table (g m^-2^).

| Species | All Ecoregions |
| --- | --- |
| CADE27 | 200 |
| PSME | 275 |
| ARME | 350 |
| LIDE3 | 550 |
| CHCH7 | 350 |
| QUGA4 | 550 |
| QUCH2 | 500 |
| QUKE | 550 |
| PIPO | 175 |
| PILA | 175 |
| PIMO3 | 250 |
| ABGRC | 200 |
| ABPRSH | 200 |
| FX_Resp_Deciduous | 550 |
| NoFX_Resp_Deciduous | 550 |
| FX_Seed_Deciduous | 550 |
| NoFX_Seed_Deciduous | 550 |
| NoFX_Resp_Evergreen | 550 |
| FX_Seed_Evergreen | 550 |
| NoFX_Seed_Evergreen | 550 |

Table S14. Maximum biomass table. Values are in g m^-2^ and were estimated from (Hudiburg *et al.*, 2009; Loudermilk *et al.*, 2013).

| Species | All Ecoregions |
| --- | --- |
| CADE27 | 30000 |
| PSME | 100000 |
| ARME | 50000 |
| LIDE3 | 60000 |
| CHCH7 | 40000 |
| QUGA4 | 30000 |
| QUCH2 | 60000 |
| QUKE | 60000 |
| PIPO | 40000 |
| PILA | 40000 |
| PIMO3 | 10000 |
| ABGRC | 60000 |
| ABPRSH | 60000 |
| FX_Resp_Deciduous | 2000 |
| NoFX_Resp_Deciduous | 2000 |
| FX_Seed_Deciduous | 2000 |
| NoFX_Seed_Deciduous | 2000 |
| NoFX_Resp_Evergreen | 2000 |
| FX_Seed_Evergreen | 2000 |
| NoFX_Seed_Evergreen | 2000 |

#

Table S15. Fire statistics according to different time periods.

|  |  | **FIRE SIZE DISTRIBUTION IN HA** | | | | |
| --- | --- | --- | --- | --- | --- | --- |
|  | **FRP (years)** | **Median**  **size** | **90^th^ percentile** | **Max size** | **N fires** | **N fires/year** |
| **Complete time series**  **(1984-2010)** | **110.516** | **1258** | **11768** | **200444** | **123** | **4.92** |
| **Complete time series**  **without Biscuit Fire**  **(1984-2010)** | 152.3627 | 1255 | 11144 | 50383 | 122 | 4.88 |
| **Time series 1**  **(1985-1995)** | 155.4146 | 1532 | 8285 | 39132 | 50 | 5 |
| **Time series 2**  **(2000-2010)** | 72.0719 | 1251 | 12155 | 200444 | 59 | 5.9 |
| **Time series2**  **without Biscuit fire**  **(2000-2010)** | **130.5128** | **1206** | **11888** | **40858** | **58** | **5.8** |

Table S16. Fire regime calibration: Fire Size distributions and Severity.

|  | Fire Size distribution | | Fire Rotation Period *^1^ | Fire  Severity *^2^ |
| --- | --- | --- | --- | --- |
|  | Median  (ha) | 90th percentile  (ha) | (years) | (% crown damage) |
| Calibration period  (2000-2010 without Biscuit Fire) | 1,206 | 11,888 | 130 | 55 |
| Simulation  (average of 10 repetitions) | 1,177 | 11,091 | 122 | 68 |

*1 Fire rotation period (FRP): number of years required to burn an area equal to the study area. This index characterizes the fire regime in terms of fire size and fire frequency.

*2 Severity was calculated as percent crown damage in a fire. These values were extracted from a relationship between remotely sensed index dNBR (MTBS, (Eidenshink *et al.*, 2007)) and the relationship between dNBR and percent crown damage estimated by Thompson et al. (2007) in the region.

Table S17. Shape parameters for fire size distributions for all climate models by fire region.

| Fire region 1 | | Fire region 2 | | Fire region 3 | |
| --- | --- | --- | --- | --- | --- |
| µ | σ | µ | σ | µ | σ |
| 3.8 | 0.072 | 7.1 | 0.58 | 7.1 | 0.58 |

Table S18. Fuel types description and parameters for the Dynamic Fire and Fuel extension of LANDIS-II.

| Description | a | b | c | q | Mean  BUI | CBH  (m) | Characteristic species |
| --- | --- | --- | --- | --- | --- | --- | --- |
| Conifer | 110 | 0.0282 | 1.5 | 0.5 | 62 | 1 | young mixed conifer |
| Conifer | 110 | 0.0282 | 1.5 | 0.7 | 64 | 2 | mid-aged mixed conifer |
| Conifer | 110 | 0.0282 | 1.5 | 0.7 | 64 | 4 | old mixed conifer |
| Conifer | 110 | 0.0282 | 1.5 | 0.5 | 62 | 1 | young pine |
| Conifer | 72 | 0.05 | 3.5 | 0.7 | 62 | 2 | mid-aged pine |
| Conifer | 72 | 0.05 | 3.5 | 0.7 | 62 | 5 | old pine |
| Deciduous | 14 | 0.12376 | 2.827 | 0.9 | 32 | 1 | young deciduous |
| Deciduous | 14 | 0.12376 | 2.827 | 0.9 | 32 | 2 | old deciduous |
| Deciduous | 14 | 0.12376 | 2.827 | 0.9 | 75 | 2 | oak |
| Conifer | 110 | 0.0282 | 1.5 | 0.7 | 64 | 1 | all evergreen shrubs |
| Conifer | 110 | 0.0282 | 1.5 | 0.7 | 64 | 1 | all [deciduous] shrubs |
| Open | 250 | 0.035 | 1.7 | 1 | 1 | 0 | late summer, fall dry grass |

**References**

Balster NJ, Marshall JD (2000) Decreased needle longevity of fertilized Douglas-fir and grand fir in the northern Rockies. *Tree Physiology,* **20**, 1191-1197.

Beach EW, Halpern CB (2001) Controls on conifer regeneration in managed riparian forests: effects of seed source, substrate, and vegetation. *Canadian Journal of Forest Research,* **31**, 471-482.

Bonner FT, Karrfalt RT (2008) *The Woody Plant Seed Manual. Agriculture Handbook 727*, U.S. Department of Agriculture, Forest Service.

Burns RM, Honkala BH (1990) Silvics of North America: 1. Conifers; 2. Hardwoods. Agriculture Handbook 654. Washington, D.C., U.S. Department of Agriculture, Forest Service. 877 p.

Chen H, Harmon ME, Griffiths RP (2001) Decomposition and nitrogen release from decomposing woody roots in coniferous forests of the Pacific Northwest: a chronosequence approach. *Canadian Journal of Forest Research,* **31**, 246-260.

Chen H, Harmon ME, Sexton J, Fasth B (2002) Fine-root decomposition and N dynamics in coniferous forests of the Pacific Northwest, USA. *Canadian Journal of Forest Research,* **32**, 320-331.

Cross A, Perakis SS (2011) Tree species and soil nutrient profiles in old-growth forests of the Oregon Coast Range. *Canadian Journal of Forest Research,* **41**, 195-210.

Dale VH, Hemstrom M, Franklin J (1986) Modeling the long-term effects of disturbances on forest succession, Olympic Peninsula, Washington. *Canadian Journal of Forest Research,* **16**, 56-67.

Debell DS, Radwan MA (1984) Foliar Chemical Concentrations in Red Alder Stands of Various Ages. *Plant and Soil,* **77**, 391-394.

Edmonds RL (1980) Litter Decomposition and Nutrient Release in Douglas-Fir, Red Alder, Western Hemlock, and Pacific Silver Fir Ecosystems in Western Washington. *Canadian Journal of Forest Research,* **10**, 327-337.

Eidenshink J, Schwind B, Brewer K, Zhu Z-L, Quayle B, Howard S (2007) Project for monitoring trends in burn severity. Fire ecology.

Entry J, Martin N, Kelsey R, Cromack Jr K (1992) Chemical constituents in root bark of five species of western conifer saplings and infection by Armillaria ostoyae. *Phytopathology,* **82**, 393-397.

Fenn ME, Haeuber R, Tonnesen GS *et al.* (2003) Nitrogen emissions, deposition, and monitoring in the western United States. *BioScience,* **53**, 391-403.

Harlow BA, Duursma RA, Marshall JD (2005) Leaf longevity of western red cedar (Thuja plicata) increases with depth in the canopy. *Tree Physiology,* **25**, 557-562.

Harrington CA (2006) Biology and Ecology of Red Alder. In: *Red alder—a state of knowledge. General Technical Report PNW-GTR-669.* (eds Deal RL, Harrington CA) pp 21-54. Portland, OR, U.S. Department of Agriculture, Pacific Northwest Research Station.

Hudiburg TW, Law BE, Thornton PE (2013) Evaluation and improvement of the Community Land Model (CLM4) in Oregon forests. *Biogeosciences,* **10**, 453-470.

Innes RJ (2013) Fire Effects Information System. U.S. Department of Agriculture, Forest Service, Rocky Mountain Research Station, Fire Sciences Laboratory (Producer). Available at: http://www.fs.fed.us/database/feis.

Law BE, Turner D, Campbell J, Sun OJ, Van Tuyl S, Ritts WD, Cohen WB (2004) Disturbance and climate effects on carbon stocks and fluxes across western Oregon USA. *Global Change Biology,* **10**, 1429-1444.

Lewis H (1950) The significant chemical components of Western Hemlock. Douglas Fir, Western Red Cedar, Loblolly Pine and Black Spruce. *Tappi,* **33**, 299-301.

Loudermilk EL, Scheller RM, Weisberg PJ, Yang J, Dilts TE, Karam SL, Skinner C (2013) Carbon dynamics in the future forest: the importance of long-term successional legacy and climate-fire interactions. Global Change Biology, n/a-n/a.

Niemiec SS, Ahrens GR, Willits S, Hibbs DE (1995) Hardwoods of the Pacific Northwest. Corvallis, OR, Forest Research Laboratory, Oregon State University.

Franklin JF, Waring RH (1980) Distinctive features of the northwestern coniferous forest: development, structure, and function. In: *Forests: Fresh perspectives from ecosystem analysis. Proceedings of the 40th Annual Biology Colloquium.* (ed Waring RH) pp 59-85. Corvallis, Oregon, Oregon State University Press.

Hobbie SE, Reich PB, Oleksyn J, Ogdahl M, Zytkowiak R, Hale C, Karolewski P (2006) Tree species effects on decomposition and forest floor dynamics in a common garden. *Ecology,* **87**, 2288-2297.

Hudiburg T, Law B, Turner DP, Campbell J, Donato D, Duane M (2009) Carbon dynamics of Oregon and Northern California forests and potential land-based carbon storage. *Ecological applications*, **19**, 163–180.

Johnson DW, Cole DW, Bledsoe CS *et al.* (1982) Chapter 7: Nutrient Cycling in Forests of the Pacific Northwest. In: *Analysis of Coniferous Forest Ecosystems in the Western United States.* (ed Edmonds RL) pp 186-232. Stroudsburg, PA, Hutchinson Ross Publishing Company.

Keenan RJ, Prescott CE, Kimmins JP, Pastor J, Dewey B (1996) Litter decomposition in western red cedar and western hemlock forests on northern Vancouver Island, British Columbia. *Canadian Journal of Botany,* **74**, 1626-1634.

Northeastern Ecosystem Research Cooperative (NERC) foliar chemistry database (2013) USDA Forest Service Northeastern Research Station and University of New Hampshire Complex Systems Research Center. Available online at http://www.folchem.sr.unh.edu.

Parton WJ, Anderson DW, Cole CV, Steward JWB (1983) Simulation of soil organic matter formation and mineralization in semiarid agroecosystems. Special Publication No. 23. In: *Nutrient cycling in agricultural ecosystems.* (eds Lowrance RR, Todd RL, Asmussen LE, Leonard RA). Athens, Georgia, The University of Georgia, College of Agriculture Experiment Stations.

Pardo LH, Robin-Abbott M, Duarte N, Miller EK (2005) *Tree chemistry database (version 1.0). Gen. Tech. Rep. NE-324,* Newtown Square PA, U.S. Department of Agriculture, Forest Service, Northeastern Research Station.

Perakis S, Maguire D, Bullen T, Cromack K, Waring R, Boyle J (2006) Coupled nitrogen and calcium cycles in forests of the Oregon Coast Range. *Ecosystems,* **9**, 63-74.

Pierce LL, Running SW, Walker J (1994) Regional-scale relationships of leaf area index to specific leaf area and leaf nitrogen content. *Ecological Applications,* **4**, 313-321.

Prescott CE, Chappell HN, Vesterdal L (2000) Nitrogen turnover in forest floors of coastal Douglas-fir at sites differing in soil nitrogen capital. *Ecology,* **81**, 1878-1886.

Prescott CE, Preston CM (1994) Nitrogen Mineralization and Decomposition in Forest Floors in Adjacent Plantations of Western Red Cedar, Western Hemlock, and Douglas-Fir. *Canadian Journal of Forest Research,* **24**, 2424-2431.

Scheller RM, Hua D, Bolstad PV, Birdsey RA, Mladenoff DJ (2011) The effects of forest harvest intensity in combination with wind disturbance on carbon dynamics in Lake States mesic forests. *Ecologisilvical Modelling,* **222**, 144-153.

Schowalter TD, Morrell JJ (2002) Nutritional quality of Douglas-fir wood: Effect of vertical and horizontal position on nutrient levels. *Wood and Fiber Science,* **34**, 158-164.

Scott EE, Perakis SS, Hibbs DE (2008) delta N-15 patterns of Douglas-fir and red alder riparian forests in the Oregon coast range. *Forest Science,* **54**, 140-147.

Short, Karen C. (2013) Spatial wildfire occurrence data for the United States, 1992-2011 [FPA_FOD_20130422]. Fort Collins, CO: U.S. Department of Agriculture, Forest Service, Rocky Mountain Research Station. https://doi.org/10.2737/RDS-2013-0009

Soil Survey Staff, Natural Resources Conservation Service, United States Department of Agriculture. Web Soil Survey. Available online at http://websoilsurvey.nrcs.usda.gov/. Accessed 03/20/2013.

Sollins P, Grier CC, Mccorison FM, Cromack K, Fogel R (1980) The Internal Element Cycles of an Old-Growth Douglas-Fir Ecosystem in Western Oregon. *Ecological Monographs,* **50**, 261-285.

Thomas KD, Prescott CE (2000) Nitrogen availability in forest floors of three tree species on the same site: the role of litter quality. *Canadian Journal of Forest Research,* **30**, 1698-1706.

Thompson RS, Anderson KH, Bartlein PJ (2000a) Atlas of relations between climatic parameters and distributions of important trees and shrubs in North America. In: *U S Geological Survey professional paper 1650 A-B.* Denver, CO, U.S. Department of the Interior, U.S. Geological Survey.

Thompson RS, Anderson KH, Bartlein PJ, Smith SA (2000b) Atlas of relations between climatic parameters and distributions of important trees and shrubs in North America - Additional Conifers, Hardwoods, and Monocots. In: *U S Geological Survey professional paper 1650C.* Denver, CO, U.S. Department of the Interior, U.S. Geological Survey.

Torregrosa A, Combs C, Peters J (2016) GOES-derived fog and low cloud indices for coastal north and central California ecological analyses: COASTAL FLC FREQUENCY. Earth and Space Science, 3, 46–67.

Tree Species Compendium. Available online at http://www.for.gov.bc.ca/hfp/silviculture/Compendium/.

Valachovic YS, Caldwell BA, Cromack K, Griffiths RP (2004) Leaf litter chemistry controls on decomposition of Pacific Northwest trees and woody shrubs. *Canadian Journal of Forest Research,* **34**, 2131-2147.

Yang Y, Luo Y (2011) Carbon : nitrogen stoichiometry in forest ecosystems during stand development. *Global Ecology and Biogeography,* **20**, 354-361.

Zhang L, Jacob DJ, Knipping EM *et al.* (2012) Nitrogen deposition to the United States: distribution, sources, and processes. *Atmospheric Chemistry and Physics,* **12**, 4539-4554.
